# Design, Fabrication and Characterization of Biodegradable Composites Containing *Closo*-Borates as Potential Materials for Boron Neutron Capture Therapy

**DOI:** 10.3390/polym14183864

**Published:** 2022-09-15

**Authors:** Mariia Stepanova, Anatoliy Dobrodumov, Ilia Averianov, Iosif Gofman, Juliya Nashchekina, Ivan Guryanov, Ilya Klyukin, Andrey Zhdanov, Evgenia Korzhikova-Vlakh, Konstantin Zhizhin

**Affiliations:** 1 Institute of Macromolecular Compounds, Russian Academy of Sciences, St. Petersburg 199004, Russia; 2 Institute of Cytology, Russian Academy of Sciences, St. Petersburg 194064, Russia; 3 Institute of Chemistry, Saint-Petersburg State University, St. Petersburg 198504, Russia; 4 Kurnakov Institute of General and Inorganic Chemistry, Russian Academy of Sciences, Moscow 119991, Russia

**Keywords:** *closo*-borates, aliphatic polyesters, poly(lactic acid), poly(ε-caprolactone), alginate/gelatin hydrogel, biocompatible 2D and 3D polymer composites, mechanical and degradation properties, drug delivery system for boron neutron capture therapy

## Abstract

Boron neutron capture therapy (BNCT) has been recognized as a very promising approach for cancer treatment. In the case of osteosarcoma, boron-containing scaffolds can be a powerful tool to combine boron delivery to the tumor cells and the repair of postoperative bone defects. Here we describe the fabrication and characterization of novel biodegradable polymer composites as films and 3D-printed matrices based on aliphatic polyesters containing *closo*-borates (CB) for BNCT. Different approaches to the fabrication of composites have been applied, and the mechanical properties of these composites, kinetics of their degradation, and the release of *closo*-borate have been studied. The most complex scaffold was a 3D-printed poly(ε-caprolactone) matrix filled with CB-containing alginate/gelatin hydrogel to enhance biocompatibility. The results obtained allowed us to confirm the high potential of the developed composite materials for application in BNCT and bone tissue regeneration.

## 1. Introduction

The creation of targeted drug delivery systems and their activation exclusively at the site of the pathological process is one of the main requirements of modern personalized medicine. Unlike traditional medicinal therapy, targeted drug delivery provides more efficient treatment and does not affect other organs and healthy cells [1,2]. This is particularly important in the case of oncology diseases, where the dosage of anticancer drugs used for systemic anticancer therapy exceeds the toxicity limits for healthy tissues. Strategies to address the low selectivity of conventional anticancer drugs include local drug delivery, monoclonal antibody therapy, molecularly targeted cancer therapy, and nanomedicine [3,4,5,6]. However, these approaches have many disadvantages while not completely solving the problem of side effects. Therefore, attempts continue to address the problem of low selectivity of antitumor treatment.

Boron neutron capture therapy has been recognized as an advanced radiation technique for cancer treatment at the cellular level [7]. It is based on the nuclear capture and ^10^B(n,α)^7^Li fission reaction, when non-radioactive boron-10 is irradiated with low energy thermal neutrons with the formation of the ^11^B isotope and its instant decay to high linear energy transfer α-particle (^4^He) and recoiled lithium-7 (^7^Li) nucleus with the production of low linear energy transfer γ-radiation. Since the forming particles (^4^He and ^7^Li) have very short path lengths in tissue (<10 µm), their destructive effects are limited to boron-containing cells. The minimum concentration of ^10^B required for lethal cell damage is about 1 × 10^9 10^B atoms per cell [8]. The most important requirements for BNCT delivery agents are (1) low intrinsic toxicity, (2) high tumor uptake (~20 μg/g of tissue) and low normal tissue uptake, preferably with tumor-normal tissue and tumor-blood boron concentration ratios of not less than 3:1, and (3) relatively rapid clearance from blood and normal tissues and persistence in the tumor during BNCT [9]. Accordingly, many compounds with high boron content to achieve the necessary ^10^B concentration in the cancer cells were proposed for BNCT. Among them, sodium mercapto-undecahydro-*closo*-dodecaborate (sodium borocaptate, Na_2_[B_12_H_11_SH]) and 4-dihydroxy-borylphenylalanine (boronophenylalanine, BPA) have found clinical applications due to their sufficient biodistribution profiles, toxicology, and therapeutic effect [10]. However, though initial results with BNCT are very promising, toxicity remains relatively high, and selective delivery of boron agents to the tumor sites to decrease potential toxicity is still a critical issue that impairs the further clinical development of this method [11]. To afford tumor cell specificity and targeted delivery of BNCT agents, various third-generation boron-containing systems were developed on the base of immunoliposomes, boronated co-polymers, linear and cyclic peptides, DNA intercalators, EGF/anti-EGFR monoclonal antibodies, porphyrins, etc. [12,13,14,15,16,17,18].

BNCT can be an efficient and safe method to treat radio-resistant types of tumors, such as aggressive bone cancer, osteosarcoma [19,20]. The standard treatment of this malignancy includes a combination of chemotherapy and surgical removal of all detectable tumor foci, which can lead to large bone defects and to the loss of bone strength with consecutive patient disability and a decrease in the quality of life [21]. Thus, in the case of osteosarcoma, not only the problem of the risk of tumor recurrence has to be eliminated, but also normal bone functioning and bone defect repair have to be ensured.

Various techniques were proposed to repair the damaged bone, such as autologous, allogenic, and synthetic bone grafting, artificial joint replacement, and the induced membrane approach [22]. Synthetic organic and inorganic bone substitutes have many advantages since the implanted material can function as a biodegradable scaffold, which can afford temporary support for the cells and promote cell differentiation into the bone by additional immobilization and release of various exogenous signaling molecules and facilitate the repair of bone defects. The biomaterial used for bone regeneration has to meet many requirements, such as biocompatibility, low immunogenicity, osteoconductivity, and osteoinductivity. It also has to have sufficient mechanical strength and porosity and stimulate vascularization [22,23,24,25,26]. It is necessary for normal angiogenesis and healthy bone tissue formation during the gradual biodegradation of the scaffold.

Among synthetic polymers, poly(ε-caprolactone) (PCL), polylactide, poly(lactic-*co*-glycolic acid), poly(vinyl alcohol), poly(ethylene glycol), and their copolymers are the most used as supports for bone regeneration due to their excellent biocompatibility, tailored structural and mechanic characteristics, and controllable degradation rate, which is important for the gradual substitution of the polymer scaffold by de novo forming bone tissue [27,28,29,30,31]. To additionally improve the biological safety for clinical applications and accelerate tissue regeneration by the involvement of various signaling molecules and growth factors, composite osteogenic scaffolds with bio-based polymers, such as collagen, cellulose, chitosan, and hyaluronic acid, were developed [32,33,34].

From this point of view, the incorporation of the BNCT agents in such implantable polymer-based delivery systems, which can release boron-containing compounds in the proximity of residual tumor foci, can be a promising approach for the postoperative treatment of cancer to prevent metastasis of the nearest tissues. Interestingly, no implantable biomaterials as local delivery systems for BNCT agents have been described so far for the treatment of osteosarcoma. Here, we studied the possibility of the application of biodegradable and biocompatible aliphatic polyesters in the form of films or 3D-printed matrices for the immobilization of boron-rich *closo*-borates, suitable for BNCT. In the last case, the polymer support was also filled with alginate/gelatin hydrogel to increase the biocompatibility of the material and the rate of bone tissue repair. Surface morphology, mechanical properties, the kinetics of material degradation and CB release in model physiological conditions, and biological properties in vitro for the composites were examined and compared. Thus, the approach proposed in this work can afford a combination of local anticancer drug delivery and the regeneration of the damaged bone after BNCT and/or surgical resection, and improve the existing methods of osteosarcoma treatment.

## 2. Materials and Methods

### 2.1. Chemicals and Materials

All monomers, initiators, reagents for the synthesis of poly(amino acids) and the removal of the protective groups, tin (II) octoate (Sn(Oct)_2_), lithium bromide, lipase from *Candida rugosa* (1302 units/mg), papain from papaya latex (13 units/mg), 3-trimethylsilyl-1-propanesulfonic acid sodium salt (DSS), 15-crown-5 (crown ether) were purchased from Sigma-Aldrich (Darmstadt, Germany). Organic solvents, salts for the preparation of buffer solutions, sodium hydroxide, hydrochloric acid, calcium chloride anhydrous, boric acid, ninhydrin, and ethylenediamine used in this work with a purity of more than 99% were products of Veckton Ltd. (St. Petersburg, Russia). All solvents were distilled prior to application. Deuterium oxide and deuterated acetonitrile were products of Solvex LLC (Moscow, Russia).

Ultrapure water (18.2 MΩ·cm, Milli-Q system, Millipore, Bedford, MA, USA), HNO_3_ (69%, Merck, Darmstadt, Germany), and single-element standard stock solution containing 1000 mg/L B (Agilent Technologies, Santa-Clara, CA, USA) were used to prepare working and standard reference solutions for the determination of boron in films with *closo*-borates adsorbed on their surface.

Gelatin from pig skin (type A, 270 Bloom, 20 mesh) and very low viscosity alginic acid sodium salt, used to form the containing *closo*-borate hydrogel to fill the macropores of a 3D-printed PCL-based matrix, were purchased from Trobas Gelatine BV (Dongen, NL, USA) and Alfa Aesar (Heysham, UK), respectively.

Human MG-63 osteosarcoma cells were cultivated in Eagle’s minimum essential medium (EMEM; Lonza, St. Louis, MO, USA) with an addition of 1% non-essential amino acids (NEAA; Gibco, Grand Island, NY, USA), 10% fetal bovine serum (FBS; HyClone, Logan, UT, USA) and 1% streptomycin/penicillin (Sigma-Aldrich, Darmstadt, Germany) and cultivated in a humidified 5% CO_2_ at 37 °C. This cell line was provided by the Vertebrate Cell Culture Collection of the Institute of Cytology RAS (St. Petersburg, Russia). In the experiments, 4–6 passages cells were used. At 80–90% confluence, the cells were detached with trypsin-EDTA (Sigma-Aldrich, Darmstadt, Germany).

### 2.2. Synthesis of Closo-Borates

Sodium salts of substituted *closo*-borates were prepared from pre-synthesized accordingly to known procedures (NBu_4_)[B_12_H_11_NH_3_] [35], (NBu_4_)_2_[B_10_H_9_OH] [36], (NBu_4_)[B_10_H_9_NH=C(OH)CH_3_] [37], and (NBu_4_)[B_10_H_9_NH=C(NH_2_)CH_3_] (obtained similarly to (NBu_4_)[B_12_H_11_NH=C(NH_2_)CH_3_] *closo*-dodecaborate [38]) by cation metathesis reaction with Na[BPh_4_], as described in [38]. The completeness of the reaction was monitored using ^11^B NMR-spectroscopy. After the disappearance of the signal of [BPh_4_]^−^ anion in the aqueous fraction of the water–organic emulsion, the reaction was stopped. ^1^H, ^13^C, and ^11^B NMR spectra of the CB were performed at the Resource Center of the Kurnakov Institute of General and Inorganic Chemistry RAS on a Bruker Avance II 300 spectrometer (Bruker, Karlsruhe, Germany). Tetramethylsilane and boron trifluoride ether were used as the external standards. (NBu_4_)[B_10_H_9_NH=C(NH_2_)CH_3_], ^11^B NMR (CD_3_CN), *δ*, ppm: 2.3 (d, 1B, B(10), J(B-H) = 153 Hz), –4.3 (d, 1B, B(1), J(B-H) = 144 Hz), −15.0 (s, 1B, B(2)), –24.1 (d, 4B, B(3,5,6,9), J(B-H) = 123 Hz), –27.3 (d, 3B, B(4,7,8), J(B-H) = 128 Hz); ^1^H NMR (CD_3_CN), δ, ppm: −1.01–1.55 (m, 9H, B_10_H_9_), 7.91 (br.s, 1H, C-NH_2_), 6.63 (br.s, 1H, C-NH_2_), 6.33 (br.s, 1H, C=NH), 3.15 (m, 8H, NBu_4_), 2.00 (s, 3H, NH=CH_3_), 1.66 (m, 8H, NBu_4_), 1.42 (m, 8H, NBu_4_), 1.02 (t, 12H, NBu_4_); ^13^C NMR (CD_3_CN), δ, ppm: 165.7 (C=NH), 58.4 (NBu_4_), 23.3 (NBu_4_), 19.7 (CH_3_-C=NH), 19.3 (NBu_4_), 12.8 (NBu_4_). The yield was about 78%.

The ^11^B NMR spectra of Na[B_12_H_11_NH_3_]·2H_2_O, Na_2_[B_10_H_9_OH]·2H_2_O, Na[B_10_H_9_NH=C(NH_2_)CH_3_]·2H_2_O, and Na[B_10_H_9_NH=C(OH)CH_3_]·2H_2_O are shown in Figure S1 (Supplementary Materials).

Thermal properties of CB were studied using a synchronous (TGA/DSC) thermal analyzer SDT Q600 (TA Instruments, New Castle, DE, USA). About 6 mg of the sample was placed in an Al_2_O_3_ microcrucible and heated to 300 °C at a rate of 10 °C/min in a flow of argon (99.995%) or dry air (100 mL/min).

### 2.3. Synthesis and Characterization of Polymers

The synthesis of poly-*D*,*L*-lactide (PDLLA), and PCL was carried out in bulk by ring-opening polymerization (ROP) of the corresponding monomers (*D*,*L*-lactide, and *ε*-caprolactone). The reaction conditions were optimized to obtain polymers with similar molecular weight characteristics and intrinsic viscosities. Polymerization was carried out at 130 °C for 4 h under vacuum for PDLLA and for 21 h under argon atmosphere for PCL. The monomer to tin (II) octoate molar ratio in the case of PDLLA synthesis was 2500. PCL was synthesized by adding methanol as a co-initiator to the reaction system, and the ratio of [monomer]:[Sn(Oct)_2_]:[MeOH] was 5000:1:2. The resulting polymers were dissolved in a minimum amount of chloroform, precipitated into methanol, filtered, washed 2 times with methanol, and dried in vacuum at ~150 Pa. PDLLA and PCL yields were 83 and 57%, respectively.

Poly(*L*-lysine) (PLys) and random poly(amino acids), e.g., poly((*L*-lysine)-*co*-(*L*-leucine)) (P(Lys-*co*-Leu)) and poly((*L*-lysine)-*co*-(*L*-phenylalanine)) (P(Lys-*co*-Phe)), used to modify the surface of films based on aliphatic polyesters with amino groups were obtained at the Institute of Macromolecular Compounds RAS according to the previously developed protocol [39,40]. The synthesis was carried out by ROP of pre-synthesized N-carboxyanhydrides (NCA) of the corresponding α-amino acids. *n*-Hexylamine was used as the initiator. The molar ratio of monomer to initiator was 50. The molar ratio between *ε*-benzyloxycarbonyl-*L*-lysine (Lys(Z)) NCA and the hydrophobic amino acid NCA was 4 in the case of *L*-leucine NCA and 8 in the case of *L*-phenylalanine NCA.

Weight average molecular weights (*M_w_*) and dispersity (*Ð*) of all synthesized polymers were determined by size-exclusion chromatography (SEC) using a Shimadzu HPLC system (Tokyo, Japan) consisting of a pump LC-10AD VP, refractometric detector RID-10A, column oven CTO-20A, system controller SCL-10A VP (Canby, OR, USA) supplied with a Rheodyne 725i injection valve (Rohnert Park, CA, USA) and two Agilent PLgel MIXED-D columns (5 µм, 7.5 × 300 mm, Agilent Technologies, Santa-Clara, CA, USA). LC Solution Shimadzu software (version 1.25, Kyoto, Japan) was used to collect and process data.

Molecular weight characteristics of aliphatic polyesters were determined at a mobile phase flow rate of 1.0 mL/min and 40 °C using tetrahydrofuran as an eluent and polystyrene standards with molecular weights ranging from 2000 to 450,000 (Agilent Technologies, Santa Clara, CA, USA and Waters, Milford, MA, USA) for calibration. *M_w_* and *Ð* for PDLLA and PCL were 137,000, 1.7, and 123,000 and 1.6, respectively. In the case of poly(amino acids), the analysis was carried out for protected (co)polymers using 0.1 M solution of lithium bromide in *N*,*N*-dimethylformamide as a mobile phase and poly(methyl methacrylate) standards with molecular weights ranging from 17,000 to 250,000 for calibration at a mobile phase flow rate of 0.7 mL/min and 40 °C. Their molecular weight characteristics were as follows: *M_w_* = 38,500 and *Ð* = 1.1 for PLys(Z); *M_w_* = 26,600 and *Ð* 1.3 for P(Lys(Z)-*co*-Leu); *M_w_* = 36,300 and *Ð* = 1.4 for P(Lys(Z)-*co*-Phe). Deprotection of poly(amino acids) was carried out with the use of a 5.25% solution of trifluoromethanesulfonic acid in trifluoroacetic acid for 4 h. The complete deprotection was confirmed by ^1^H NMR spectroscopy (AVANCE AV-400 NMR spectrometer, Bruker, Karlsruhe, Germany) by the disappearance from the spectra of signals corresponding to the methylene group (5.0 ppm) and the aromatic fragment (7.1 ppm) of Z-protective groups (-O-CH_2_-C_6_H_5_).

The viscosity of a series of aliphatic polyesters solutions at various concentrations in chloroform was measured using an Ostwald’s capillary viscosimeter. Intrinsic viscosities calculated from the obtained data were 1.1 and 1.3 dL/g for PDLLA and PCL, respectively.

### 2.4. Manufacturing of Films

The fabrication of polymer pristine (as control) and composite films with a thickness of 90 ± 10 μm was carried out as described in [41]. Namely, 6.8 mL of 5 wt% PDLLA/PCL solution in chloroform was poured into a glass ring (78 mm i.d.) with cellophane on the bottom and dried for 12–15 h at room temperature in air. Then the cellophane support was removed, and the films were dried from chloroform residues for 2 weeks (PDLLA) and 1 week (PCL) at 40 °C in an air thermostat.

In the case of composite films, a suspension of the filler in a polymer solution was prepared using the ultrasound homogenizer Sonopuls MS 73 (Bandelin, Berlin, Germany) for 30 s at 60–70% power. The casting and drying processes to obtain filled films were the same as described above. Copolymers of amino acids (10 wt%), namely P(Lys-*co*-Leu) and P(Lys-*co*-Phe), and Na[B_12_H_11_NH_3_]·2H_2_O (1, 3, and 5 wt%) were used as fillers. The indicated amounts of filler were calculated relative to the polymer content in the solution.

### 2.5. Modification of PDLLA- and PCL-Films Surface

The surface modification of polymer films based on PDLLA and PCL was carried out in two ways: by the generation of carboxyl and hydroxyl groups and by the generation of amino groups.

Carboxyl and hydroxyl groups were obtained according to the procedures described in [42,43] by treating the film specimen with 0.1 M NaOH in a water–ethanol mixture with a ratio of 7/3 (*v*/*v*) for 30 min, followed by washing in turn with water, 0.1 M HCl aqueous solution, and again with water. These reaction conditions ensure the integrity of the film material and obtain the highest content of carboxyl groups [42].

Amino groups were introduced into the surface of polymer materials by covalent modification of partially hydrolyzed films with amino-containing compounds (ethylenediamine or PLys) using the method of activated esters. To this aim, the film specimen containing -COOH and -OH groups, generated as described above, were placed in a vial and N-hydroxysuccinimide (0.026 mol, 5 mL) and 1-ethyl-3-(3-dimethylaminopropyl)carbodiimide hydrochloride (0.013 mol, 1 mL) dissolved in 0.05 M 2-(N-morpholino)ethanesulfonate buffer solution (pH 5.6) were added. The reaction of an activated ester formation was carried out for 1 h at a temperature of 0–5 °C. After that, the films were washed twice with 0.01 M borate buffer solution (pH 8.5) and placed in solutions of ethylenediamine (22.5 mg, 8 mL including 0.5 mL 1 M HCl to adjust pH to 8.5) or PLys (11.2 mg, 5 mL) prepared using the same buffer system. The reaction was performed at 25 °C for 24 h.

Modifications were carried out using film specimen punched in the form of discs with a diameter of 15 mm. The amounts of reagents are given based on the modification of 1 disc-shaped film. After the reaction, all films were washed abundantly with water and dried in an air thermostat at 40 °C to constant weight (2–3 days).

### 2.6. Study of Adsorption/Desorption Processes of Closo-Borates

The deposition of *closo*-borates due to non-covalent interactions (adsorption) on the surface of unmodified and modified as described above film specimen, as well as the study of the kinetics of CB release (desorption), was carried out directly in NMR tubes for online monitoring of these processes by ^11^B NMR spectroscopy. NMR control and data processing were performed using an AVANCE AV-400 NMR spectrometer (Bruker, Karlsruhe, Germany) and TopSpin 2.1 software (Bruker BioSpin, Rheinstetten, Germany), respectively. For both types of experiments, two specimens of accordion-folded film discs were placed in the NMR tube. *Closo*-borates with different substituents were tested: Na[B_12_H_11_NH_3_]·2H_2_O, Na_2_[B_10_H_9_OH]·2H_2_O, Na[B_10_H_9_NH=C(NH_2_)CH_3_]·2H_2_O, and Na[B_10_H_9_NH=C(OH)CH_3_]·2H_2_O.

To assess the degree of *closo*-borate adsorption, the films preliminarily degreased with ethanol and dried at 40 °C were placed in a solution with various concentrations of CB (4 or 60 μmol/mL). D_2_O was used as a solvent. In the case of Na[B_10_H_9_NH=C(NH_2_)CH_3_]·2H_2_O and Na[B_10_H_9_NH=C(OH)CH_3_·2H_2_O], 2 vol% CD_3_CN was added to the system due to their low solubility. The experiment was carried out at 25 °C. The process of *closo*-borates adsorption was also investigated in the presence of an equimolar amount of crown ether (15-crown-5) capable of selective binding of Na^+^.

The study of the *closo*-borate desorption from the surface of the polymeric material was carried out at 25 °C in a solution simulating physiological conditions: 0.01 M phosphate buffer solution (pH = 7.4) containing 0.15 M sodium chloride (PBS). H_2_O/D_2_O in the ratio 10/90 (*v*/*v*) was used as a solvent for the preparation of the model mixture.

The solution volume in all cases was 0.5 mL. Boric acid (100 μmol/mL) was added to the NMR tube as a reference and concentration standard for determining the content of *closo*-borate in solution. Monitoring of changes in the relative concentration of CB over time was carried out using the integral intensity of signals in the ^11^B NMR spectra at the following chemical shifts (ppm): 19.80 (s, 1B) for H_3_BO_3_, −14.90 (d, 5B) and −15.75 (d, 5B) for Na[B_12_H_11_NH_3_], −24.59 (d, 4B) for Na_2_[B_10_H_9_OH], −26,46 (d, 4B) for Na[B_10_H_9_NH=C(NH_2_)CH_3_], −26.98 (d, 4B) for Na[B_10_H_9_NH=C(OH)CH_3_].

### 2.7. Manufacturing of 3D-Printed Macroporous Matrices

Pristine and composite 3D macroporous matrices based on PCL were fabricated by FDM (fused deposition modeling) 3D printing using a BioScaffolder 3.2 3D printer (Gesim, Radeberg, Germany) equipped with a pneumatic extruder and a heating stage. The GeSim Robotics software version 1.16.0.3892 (GeSim, Radeberg, Germany) was used to create 3D models and control the printer. PCL or PCL with 5 wt% *closo*-borate was loaded into a pneumatic extruder cartridge of a printer with a print head diameter of 0.4 mm. After setting the cartridge temperature, a pause of 20 min was maintained before printing began. Specimens were printed at room temperature (22 °C) using a glass substrate heated to 35 °C. The print mode was pre-optimized to ensure uniform extrusion of the material and obtain specimens of a homogeneous structure. Thus, the optimal printing parameters were as follows: pressure—540 kPa, cartridge temperature—76 °C, height of one printed layer—0.36 mm, print head speed—0.2 mm/s, distance from the print head to the printed layer—0.35 mm. The printhead movement pause at the start and at the end of the layer print was set to 4.0 and 5.0 s, respectively. The pause after printing each layer was 1 min. The printing of each subsequent layer was carried out with a rotation relative to the plane of the previous one by 60° to ensure the formation of interpenetrating pores. The specimen model was a porous cylinder with a diameter of 3.2 mm and a height of 2.5 mm, consisting of 7 layers. Each printed layer resembled the letter “s”. The lift-off motion of the print head from the printed layer was set in the horizontal direction for all specimens to avoid detachment of the entire specimen or the last straight section of the track. According to the simulated data, the volume of material spent on printing, the external geometric volume, the pore volume, and the porosity of one specimen were 0.0075 cm^3^, 0.0203 cm^3^, 0.0128 cm^3^, and 63%, respectively. The weight of the manufactured product was 11.9 ± 0.9 mg.

### 2.8. Fabrication of 3D-Printed Matrices with Closo-Borate-Containing Hydrogel

The formation of a hydrogel carrier of *closo*-borate was carried out directly in the pores of preliminarily 3D-printed macroporous PCL-based matrices, obtained as described in the previous section. Before hydrogel formation, 3D-printed matrices were degreased with ethanol and dried at room temperature. Gelatin (83.0 mg/mL) was added to a suspension of *closo*-borate (61.0 mg/mL) heated to 50 °C in an aqueous solution of alginic acid sodium salt (27.5 mg/mL) under stirring on a heated magnetic stirrer MR Hei-Standard (Heidolph, Schwabach, Germany). After gelatin dissolution, the resulting mixture was stirred at 50 °C for 20 min. Then, the printed macroporous matrices were dipped into the suspension and cooled to room temperature with stirring at a speed of 500 rpm. After 20 min, an aliquot of a 20 wt% aqueous solution of CaCl_2_ was added to the system (based on 11.3 mg of anhydrous calcium chloride per 1 g of final hydrogel), and the stirring speed was reduced to 200 rpm. Stirring was carried out for 5 min and turned off. The resulting system was incubated at room temperature for 20 min, the composite matrices were taken out, and the excess hydrogel was cut off with a scalpel, leaving it only in the intrapore space of the materials. The resulting hydrogel contained 5 wt% *closo*-borate, and the ratio of sodium alginate to gelatin was 25/75% (wt/wt). The weight of the hydrogel formed inside one matrix was 10.5 ± 0.7 mg, which corresponded to 2.0 ± 0.2 mg of the dried composite gel.

The degree of re-swelling of the gel containing 5 wt% CB was evaluated. To this aim, 1 mL of water was added to several specimens (2.5 ± 0.4 mg each specimen) preliminarily dried using a FreeZone 1L freeze dryer (Labconco, Kansas City, MO, USA) and placed in a separate container, incubated at room temperature and weighed. The degree of re-swelling was calculated by the equation:

Re-swelling degree (%) = (m/m_0_) × 100

where m_0_ is the mass of the initially obtained hydrogel with CB (mg) and m is the mass of the gel containing CB (mg) swollen after freeze-drying. The experiment was repeated three times.

### 2.9. Characterization of Materials

Tensile properties were studied in the uniaxial tension mode with the use of a Shimadzu AG-100kNX Plus universal mechanical tests system (Shimadzu, Kyoto, Japan) using film strips of 20 × 2 mm and a thickness of 90 ± 10 μm. The extension speeds were 10 mm/min for PDLLA and 50 mm/min for PCL. The characteristics of the tested films were determined and averaged according to the results of measurements on 7–9 specimens.

The mechanical properties of composites in compression were investigated for PCL-based materials using cylinder-shaped monolithic specimens with a diameter of 9 mm and a height of 4 mm. Monolithic matrices were obtained by mixing the components, homogenizing them in the polymer melt, and hot molding the prepared composite at 105 °C. Testing was carried out in uniaxial compression mode at a compression speed of 10 mm/min using a Shimadzu AG-100X Plus universal machine (Kyoto, Japan). Three specimens of each composition were tested and the results were averaged. Compression was performed to a maximum deformation of 80%. All mechanical tests were carried out at room temperature.

The presence of adsorbed *closo*-borates on the surface of the film materials was determined by attenuated total reflection-Fourier transform infrared (ATR-FTIR) spectroscopy (IR Affinity-1 S Shimadzu spectrometer, Shimadzu Corp., Kyoto, Japan). ATR-FTIR spectra were recorded from 800 to 4000 cm^−1^ at a resolution of 2 cm^−1^ using the Pike Miracle Ge ATR accessory (Pike Technologies, Madison, WI, USA) installed in the spectrometer. The content of adsorbed CB was evaluated by ^11^B NMR spectroscopy (description is given in Section 2.6). The determination of the total B amount on the surface of these materials was performed using a microwave plasma-atomic emission spectrometer (MP-AES, Agilent 4210 MP-AES, Agilent Technologies, Santa-Clara, CA, USA) equipped with a Halo nitrogen generator (Peak Scientific, Inchinnan, Scotland, UK) and with the following instrument and method parameters: HF-resistant polymer cyclonic spray chamber, axial plasma viewing, peristaltic pump speed—15 rpm, plasma stabilization time—15 s. The spectra of B were processed using the Agilent MP Expert software (version 1.6.1.10384, Agilent Technologies, Santa Clara, CA, USA) with automatic background correction. The analyzed film samples in the amount of two specimens per analysis with a total weight of 32–36 mg were placed into a PTFE reaction vial and dissolved in 10 mL of HNO_3_/water in the ratio 1/1 (*v/v*). The resultant liquid was analyzed with a linear range from 0 to 100 mg/L (calibration curve correlation coefficient is 0.9995). Experiments were carried out in triplicate using the B 249.772 nm wavelength for analysis. The specimen in the case of all described studies was a film in the form of a disk of the same size with a diameter and thickness of 15 mm and 90 ± 10 μm, respectively.

The morphology of composite and pristine polymer specimens, both in the form of films and in the form of 3D matrices, was examined by optical and scanning electron microscopy (SEM) using Nikon eclipse E200 (Tokyo, Japan) and Zeiss Auriga or Zeiss Merlin (detection of secondary electrons, Oberkochen, Germany) microscopes, respectively.

### 2.10. Investigation of Closo-Borate Release and Material Degradation

The release of *closo*-borates from the PDLLA/PCL films and scaffolds as well as polymer degradation were studied in a model physiological medium, namely PBS (0.01 or 0.1 M sodium phosphate buffer, pH 7.4, with 0.15 mol/L NaCl) containing lipase (2.5 mg/mL). The experiment was carried out directly in NMR tubes to monitor the progress of these processes using ^1^H and ^11^B NMR spectroscopy. To this aim, a specimen (3 × 40 mm strip, 10.7 ± 0.9 mg) or a 3D-printed matrix (see Section 2.7) was placed in an NMR tube with PBS solution containing lipase (0.6 mL) and incubated in an orbital thermoshaker Unimax 1010 (Heidolph, Schwabach, Germany) at 37 °C and 130 rpm with periodic NMR monitoring. Boric acid (100 μmol/mL) and DSS (4 μmol/mL) were added to the test solution as reference standards for ^11^B and ^1^H NMR spectroscopy, respectively, to calculate the accumulation of CB and lactic acid/6-hydroxycaproic acid during CB release and PDLLA/PCL degradation.

The study of the *closo*-borate release from hydrogel matrices was carried out under similar conditions, using papain (0.1 mg/mL) instead of lipase. Two specimens of matrices containing freshly synthesized hydrogel with the parameters specified in Section 2.8 were placed in an NMR tube with a model solution containing boric acid as a standard. In this case, only ^11^B NMR spectra were recorded.

In all cases, H_2_O/D_2_O in a ratio of 10/90% (*v*/*v*) was used as a solvent for the preparation of the model system. Software and NMR-spectrometer are described in Section 2.6. The following chemical shifts were used for the calculations. ^1^H NMR (*δ*, ppm): 2.91 for DSS (dd, 2H), signals in the range 1.23–1.48 (d, 3H) for lactic acid (PDLLA monomer), 1.32 (m, 2H), and 1.55 (m, 4H) for 6-hydroxycaproic acid (PCL monomer). ^11^B NMR (*δ*, ppm): 19.80 (s, 1B) for H_3_BO_3_, −14.90 (d, 5B), and −15.75 (d, 5B) for Na[B_12_H_11_NH_3_].

### 2.11. Biological Evaluation

MTT assay was used to assess the cell viability of MG-63 cells in the presence of both *closo*-borate and composite material and was performed as described elsewhere [44]. Sterilization of the *closo*-borate powder and composite films fixed in the plate before the start of the experiment was carried out using broad spectrum UV-light for 10 min. The prepared initial concentrated CB solution was further sterilized by filtration through a sterile MCE syringe filter (0.22 µm, Jet Bio-Filtration, Guangzhou, China). The biocompatibility of the composites was evaluated using manufactured films with different content of *closo*-borate in comparison with the corresponding unfilled polymer material. To evaluate the cytotoxicity of the *closo*-borate, the solutions with different concentrations of CB (from 4 to 1000 μg/mL) were added to the wells of a plate with the adhered cells (*n* = 3). In turn, disc-shaped specimens (6 mm in diameter) were attached to the bottom of a 96-well non-adhesive plate using medical glue (BF-6, Tula Pharmaceutical Company, Tula, Russia) (*n* = 3). The wells of the plate with cultured cells were used as a control (*n* = 3). Then, 100 µL of medium with 1 × 10^4^ MG-63 cells was added to each test sample (CB solution or film) and incubated at 37 °C with 5% CO_2_ for 3 days. At the end of this time, a solution of MTT (Sigma, St. Louis, MO, USA) was added to the wells and incubated at 37 °C with 5% CO_2_ for 2 h. After that, the solutions were removed and the resulting formazan was dissolved by adding dimethyl sulfoxide (50 μL) to each well. The absorbance of the obtained colored solutions was measured at 570 nm using a Thermo Fisher Multiscan Labsystems (Waltham, MA, USA) plate reader. The obtained data were processed and visualized using MS Excel software (Microsoft, Redmond, Washington, DC, USA).

## 3. Results and Discussion

### 3.1. Design of Composite Materials

In order to prepare biodegradable composites with *closo*-borates, PDLLA and PCL were used as matrix polymers. To ensure the correct comparison of the properties of the composite materials, PDLLA and PCL with close characteristics were synthesized by ROP of *D*,*L*-lactide, and *ε*-caprolactone, respectively, in the presence of tin(II) octoate as a catalyst. The molecular weight characteristics of the polymers used for the fabrication of composites were *M_w_* = 137,000, *Ð* = 1.7 for PDLLA, and *M_w_* = 123,000, *Ð* = 1.6 for PCL (according to SEC). The intrinsic viscosities of PDLLA and PCL solutions in chloroform, determined using an Ostwald viscometer, were found to be 1.1 and 1.3 dL/g, respectively.

**Figure 1:**
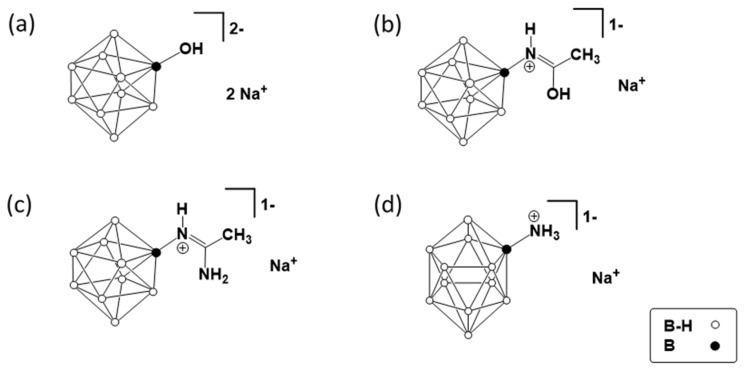
Structures of *closo*-borates: (**a**) sodium hydroxy-nonahydro-*closo*-decaborate (Na_2_[B_10_H_9_OH]), (**b**) sodium methyliminolo-nonahydro-*closo*-decaborate (Na[B_10_H_9_NH=C(OH)CH_3_]), (**c**) sodium methylamidino-nonahydro-*closo*-decaborate (Na[B_10_H_9_NH=C(NH_2_)CH_3_]), and (**d**) sodium amino-undecahydro-*closo*-dodecaborate (Na[B_12_H_11_NH_3_]).

Four *closo*-deca- (B_10_) and *closo*-dodecaborates (B_12_) suitable for the osteosarcoma treatment were used to fabricate composite materials with PDLLA and PCL. These CBs contain hydroxyl and amino groups with different acidity/basicity, which is determined by their direct or indirect bonding to the boron atoms of the cluster, as well as by in the presence of a hydrophobic environment (methyl group). Structures of CBs used are shown in Figure 1. The type of functional groups and their environment can have a significant effect on the penetration of CB into cancer cells, as well as on the interaction with the material matrix during the preparation of the composites due to the appearance of non-covalent interactions (CB adsorption). The latter is of interest for studying the peculiarities of the fabrication of such composites. The choice of CB is also determined by the presence of functional groups available for modification with bio-linkers to ensure their targeted delivery.

**Figure 2:**
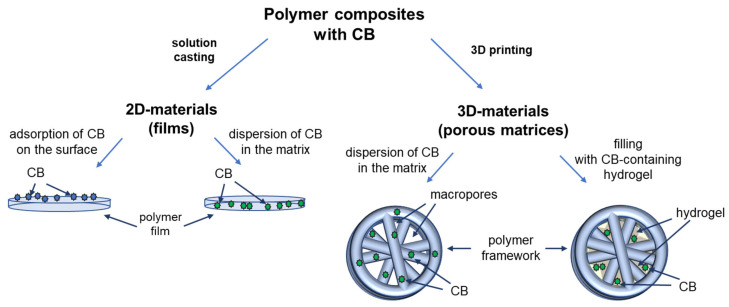
Design of the composite materials considered in the study.

Both 2D and 3D polymer materials with *closo*-borates were prepared in different ways. 2D-materials were fabricated by solution casting as films where *closo*-borates were adsorbed at the surface of the films or dispersed in the matrix. 3D-composites were manufactured via hot moulding with further 3D printing. The scheme illustrating the design of composite materials considered in this work is shown in Figure 2.

### 3.2. Polymer Films with Adsorbed Closo-Borates

#### 3.2.1. Fabrication of Films with Different Surface Functionality

Several types of PDLLA and PCL films were obtained to study the adsorption of *closo*-borates: (1) the films without any modification or additives; (2) the films containing -OH and -COOH groups on the surface; (3) PDLLA and PCL films bearing surface amino groups. Ionizable functional groups of different natures (acidic and basic) to modify the material were chosen to evaluate the most effective electrostatic interactions with *closo*-borates and ensure their easy desorption in physiological media.

The preparation of pristine PDLLA and PCL films was carried out by casting polymer solution in chloroform on the cellophane support. The resulting films were dried at room temperature and then at 40 °C to constant weight to remove residual solvent. PDLLA or PCL films with surface hydroxylic and carboxylic groups were obtained by partial alkaline hydrolysis of the corresponding initial aliphatic polyester films in the aqueous ethanol. In order to obtain the aminated surface, the generated carboxylic groups were activated to form N-hydroxysuccinimide esters, which then reacted with amine-containing low-molecular or macromolecular compound, namely ethylenediamine (EDA) or poly(*L*-lysine) (PLys, *M_w_* 38,500 and *Ð* 1.1, according to SEC). As an example, the scheme of hydrolysis and modification of PDLLA film with ethylenediamine is illustrated in Figure S2 (Supplementary Materials).

**Figure 3:**
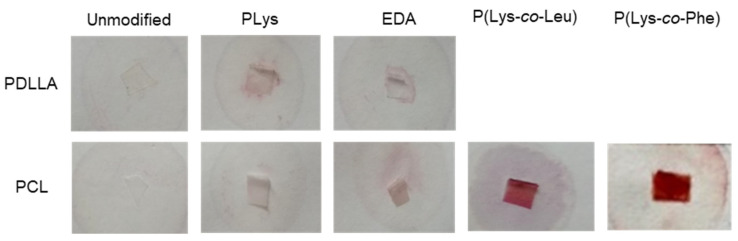
Qualitative analysis of amino groups available on the surface of PDLLA and PCL films. Staining with a 4% alcoholic solution of ninhydrin. Color intensity is proportional to the number of NH_2_-groups.

The presence of amino groups on the surface was detected using the reaction of primary amino groups with ninhydrin, which resulted in the appearance of purple color. From the images shown in Figure 3, it is seen that the staining intensity of the specimens obtained by modification with EDA or PLys is poor. This means that only a small number of amino groups were present on the surface, even in the case of PLys. To increase the number of amino groups, which are necessary for further adsorption of *closo*-borates, the biodegradable amphiphilic lysine-based copolymers P(Lys-*co*-Leu) and P(Lys-*co*-Phe) were used. In this case, functionalization of the films was achieved by distributing 10 wt% of amphiphilic polypeptides within the polymer matrix (PCL). As in the previous case, *L*-lysine was considered a source of amino groups, whereas hydrophobic *L*-phenylalanine or *L*-leucine provided an improved distribution of polypeptides in the matrix of aliphatic polyester. Analysis of the PCL-based composite films using the ninhydrin test revealed bright purple staining of composites filled with both amphiphilic polypeptides (Figure 3). Thus, the introduction of amino groups was more efficient when PCL films were filled with amphiphilic lysine-containing copolymers compared to the covalent attachment of hydrophilic lysine homopolymer at the film surface.

#### 3.2.2. Adsorption of *Closo*-Borates

The study of the adsorption was carried out by placing the film specimens into NMR tubes containing a *closo*-borate solution in D_2_O to monitor the changes in *closo*-borate concentration. Monitoring was carried out by ^11^B NMR spectroscopy using boric acid as a reference. Initially, the adsorption was studied from the solutions with a concentration of corresponding CB equal to 4 μmol/mL. Table 1 summarizes the results of the fabrication of PCL and PDLLA films with adsorbed *closo*-borates. After 10 min of incubation, the adsorption efficiency (AE) of *closo*-borate varied from 0.6% to 6.2% (Table 1), with no change during the following two weeks.

The adsorption of Na_2_[B_10_H_9_OH] on the pre-hydrolyzed surface of PDLLA showed to be two times more efficient than in the case of PCL films (3.1 wt% for PCL vs. 6.2 wt% for PDLLA). This difference can be explained by the increased number of -COOH and -OH groups formed after hydrolysis of PDLLA due to the shorter monomer chain length and the larger number of atoms linked by ester bonds at a similar molecular weight of both polymers. The adsorption of Na[B_12_H_11_NH_3_] containing an amino group at the surface of PDLLA was significantly lower (2.6 wt%). This effect may be due to the predominance of the *closo*-borate anion characteristic interaction between BH hydride hydrogen of CB and OH proton donors [45,46], accompanied by a decrease of the proton acceptor capacity from [B_10_H_10_]^2−^ to [B_12_H_12_]^2−^ [45]. At the same time, the adsorption capacity of Na_2_[B_10_H_9_OH] on the surface of PCL filled with P(Lys-*co*-Leu) (10 wt%) was slightly higher than for the same *closo*-borate adsorbed on pre-hydrolyzed PCL surface.

Since the solubility of *closo*-borates containing substituents of the amidine and iminol types in water is significantly worse than that of other *closo*-borates, D_2_O with 8 vol% CD_3_CN was used as a solvent to achieve the necessary concentration. A similar trend with better adsorption of hydroxyl-bearing *closo*-borates was observed for compounds containing hydroxyl and amino groups, which were more distant from the *closo*-borate backbone. Thus, the formation of dihydrogen bonds (proton-hydride interactions [45]) seems to be one of the main driving forces in the adsorption of CB on the surface of partially hydrolyzed aliphatic polyesters. Moreover, the presence of hydroxyl groups in the *closo*-borate structure contributed to an increase in the additional formation of classical hydrogen bonds and, thereby, to an increase in the adsorption efficiency. At the same time, both a decrease in the charge of CB anions, as well as an increase in the number of boron atoms in a cluster, leads to a decrease in the degree of CB adsorption. The presence of hydrophobic methyl groups in CB substituents did not facilitate the adsorption process.

A study of the adsorption on the surface of PDLLA and PCL films, both unmodified and modified with EDA and PLys, revealed no changes in the concentration of Na_2_[B_10_H_9_OH] after 9 days. The same result was observed for the adsorption of Na[B_12_H_11_NH_3_] at the surface of the unmodified PDLLA film. The adsorption of Na_2_[B_10_H_9_OH] from a solution with a concentration of 60 μmol/mL was also studied using specimens with various surface compositions. Despite an increase in concentration by a factor of 15, the weight adsorption capacity was retained at the same level as in the case of more diluted *closo*-borate solutions (4 μmol/mL). Higher content of amino groups in the material, as in the case of PCL containing P(Lys-*co*-Leu) (10 wt%), led to an increase in the amount of adsorbed hydroxyl-containing CB.

Thus, the adsorption of *closo*-borates on modified PDLLA and PCL materials occurs due to such non-covalent interactions as classical hydrogen and dihydrogen bonds (interaction between proton donors of materials and BH hydride hydrogen of CB). Other electrostatic interactions are weakly expressed. Stronger adsorption is observed for CB anions with a high charge and polymer fragments with a large number of more efficient proton donors—carboxyl and highly polarized hydroxyl groups of PDLLA. In the case of aminated materials, even with an increased number of protonated amino groups, the formation of this type of bonds, and therefore the degree of adsorption, is lower.

To enhance the adsorption of hydroxy-nonahydro-*closo*-decaborate on the aminated polymer surface, an equimolar amount of 15-crown-5 ether capable of selective binding of Na^+^ was added. In this case, PCL films containing P(Lys-*co*-Leu) (10 wt%) were used for the adsorption of Na_2_[B_10_H_9_OH] from the solution with a *closo*-borate concentration equal to 60 μmol/mL. The introduction of crown ether into the solution resulted in the adsorption of 39.6 ± 4.6 μg/cm^2^ or 0.219 ± 0.026 μmol/cm^2^ in 10 min. Monitoring of adsorption over the next 4 days showed no increase in adsorption of CB. Thus, the application of crown ether allowed the improvement of the adsorption capacity of hydroxyl-containing *closo*-borates at the surface of aminated PCL. This is probably due to the enhancement of electrostatic interactions between the *closo*-borate anion and the protonated amino groups of the lysine contained in the matrix.

Films containing the largest amount of CB according to ^11^B NMR data were additionally studied by MP-AES to determine the boron content. The analyzed *closo*-borate containing film specimens were obtained in a similar way, but using ultrapure water as a solvent and without adding boric acid as a standard for ^11^B NMR spectroscopy. Calculations based on elemental analysis data showed that the amounts of Na_2_[B_10_H_9_OH] adsorbed on the surface of partially hydrolyzed PDLLA and PCL/P(Lys-*co*-Leu) (preparation with the use of crown ether) films were 2.7 ± 0.7 μg/cm^2^ and 31.0 ± 2.6 μg/cm^2^, respectively. The obtained values and trends are close to those obtained using ^11^B NMR spectroscopy.

#### 3.2.3. Morphology of Prepared Materials

**Figure 4:**
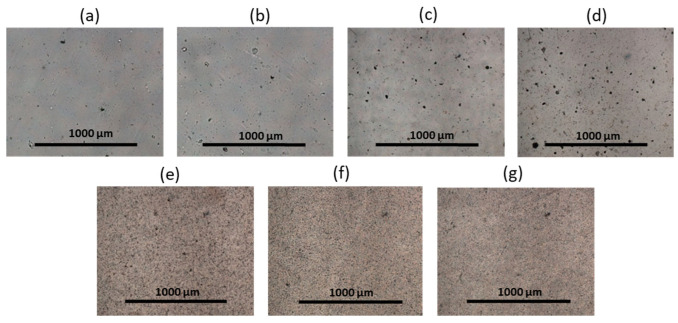
Images of polymer films obtained by optical microscopy (×4): (**a**) PDLLA, (**b**) PDLLA with carboxyl and hydroxyl groups, (**c**) PDLLA-EDA, (**d**) PDLLA-PLys, (**e**) PCL, (**f**) PCL-EDA, (**g**) PCL-PLys.

The surface morphology of the films before and after adsorption was examined using optical microscopy (magnification ×4). All films based on PDLLA before and after modification (hydrolysis, amination) remained transparent and colorless and were characterized by a homogeneous morphology (Figure 4 and Figure S3 in Supplementary Materials). However, the modification of PDLLA with EDA and PLys favored the surface roughness (Figure 4c,d). At the same time, after the adsorption of CB onto the surface of these materials, no changes in surface morphology (visualization of *closo*-borate aggregates, surface smoothing, etc.), color, and shape of the films were observed. An exception was the material based on PDLLA with carboxyl and hydroxyl groups, on the surface of which CB aggregates were detected using optical microscopy (Figure S3, Supplementary Materials).

Compared to PDLLA, polycaprolactone is known to be more hydrophobic, crystalline, and capable of forming large pores in the structure of films and other materials [41,47,48]. Indeed, most of the PCL films were characterized by porous surfaces (Figure 4e). Examination of the surface of the PCL film after partial hydrolysis and amination with EDA or PLys and subsequent adsorption of CB revealed no changes in the surface morphology (Figure 4f,g and Figure S3 in Supplementary Materials). The PCL/P(Lys-*co*-Leu) films were also characterized by a homogeneous morphology over the entire surface. These observations were confirmed by SEM (Figure S4, Supplementary Materials). In turn, the application of P(Lys-*co*-Phe) as filler to PCL was accompanied by an increase in the number of uniformly distributed pores (Figures S3 and S4, Supplementary Materials). After the adsorption of *closo*-borates, examination by optical microscopy revealed no changes in the morphology of the materials containing polypeptides as a filler.

**Figure 5:**
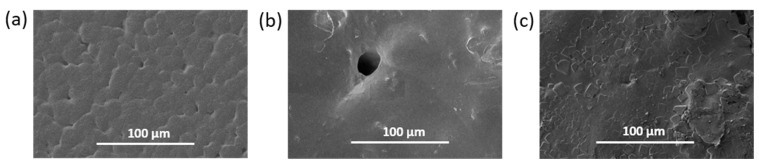
SEM images of PCL (**a**), PCL/P(Lys-*co*-Leu) (**b**), and PCL/P(Lys-*co*-Leu) with Na_2_B_10_H_9_OH adsorbed in the presence of crown ether (**c**) (×1000).

SEM investigation of the surface morphology of the PCL/P(Lys-*co*-Leu) materials based on before and after adsorption in the presence of crown ether allowed detecting many changes in the film surface morphology. The surface of the material after adsorption was characterized by greater roughness and the presence of scaly flakes in contrast to pristine PCL and initial PCL/P(Lys-*co*-Leu) films (Figure 5). This effect may indicate the presence of *closo*-borate deposits on the film.

The obtained materials were analyzed by ATR-FTIR spectroscopy using the same analysis parameters and film sample sizes. Figure S5 (Supplementary Materials) shows selected ATR-FTIR spectra both for the *closo*-borate and the films after the adsorption of CB. The appearance in the spectrum of characteristic bands in the range 2200–2640 cm^−1^ corresponding to ν(B-H) vibrations indicated the presence of *closo*-borate in the material composition. The most prominent manifestation of B-H bond oscillations was observed for the PCL/P(Lys-*co*-Leu)/Na_2_[B_10_H_9_OH] film prepared with the use of crown ether. A slightly lower signal intensity was detected for pre-hydrolyzed PDLLA/Na_2_[B_10_H_9_OH]. For all other materials after adsorption, the signal was either absent or very poorly detectable in that area. The results obtained are in agreement with ^11^B NMR spectroscopy and elemental analysis data described in Section 3.2.2, as well as SEM (Figure 5c) and optical microscopy (Figure S3g, Supplementary Materials).

### 3.3. Composite Films with Closo-Borates Dispersed in the Polymer Matrix

Another approach to preparing composite films containing *closo*-borate was based on its dispersing in the polymer matrix. In this case, a series of PDLLA and PCL composite films filled with 1, 3, or 5 wt% of *closo*-borate was obtained. The films of 90 ± 10 μm thickness were also obtained by solution casting and subsequent drying to remove the chloroform used as a solvent (see Section 2.4). In this case, Na[B_12_H_11_NH_3_] was used as a model *closo*-borate. The amine-containing CBs as promising agents in BNCT are particularly interesting because of the easiness of their conjugation. To compare the properties of the obtained composites, the polymer films without the addition of CB were fabricated in a similar way and used as control materials.

**Figure 6:**
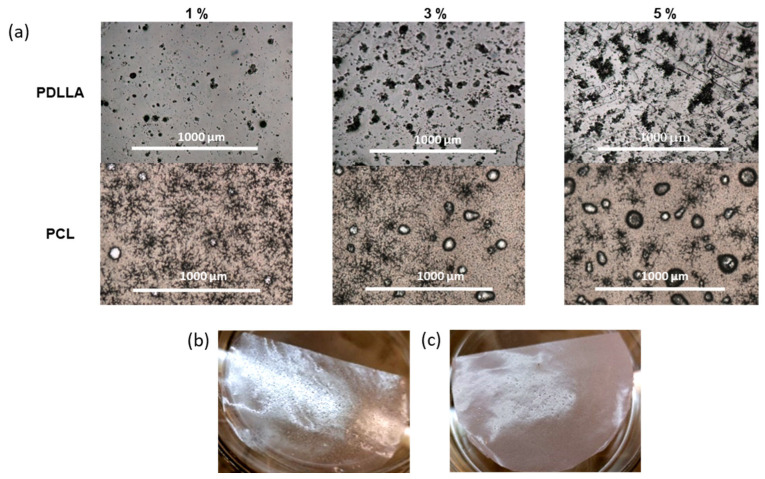
Images of composite films: optical microscopy (×4) for PDLLA and PCL films filled with different amounts of Na[B_12_H_11_NH_3_] (**a**) and photos of materials filled with 5 wt% of Na[B_12_H_11_NH_3_] based on PDLLA (**b**) and PCL (**c**).

The morphology of the obtained composite materials was examined using optical microscopy (Figure 6). In this case, Na[B_12_H_11_NH_3_] aggregates were detected for all PDLLA materials. Visual assessment and microscopic results showed that with an increase in the proportion of the filler, the size, the number of aggregates, and, accordingly, the roughness of the surface increased while their surface population was fairly uniform. Films with the addition of the filler retained their transparency and colorlessness. No particular structural changes were observed. In addition, the surface of the PDLLA ad PCL composites with Na[B_12_H_11_NH_3_] was examined by SEM (Figure S6, Supplementary Materials). The results obtained for PDLLA were in agreement with the optical microscopy data.

When *closo*-borate was added to PCL, a formation of a large number of bubbles was observed. The amount of the bubbles increased with increasing filler concentration (Figure 6). However, SEM examination of the surface did not allow the detection of *closo*-borate aggregates on the surface of the PCL materials (Figure S6, Supplementary Materials).

### 3.4. 3D-Composite Materials Containing Closo-Borates

Three-dimensional macroporous *closo*-borate-containing matrices were produced by 3D printing using a printer equipped with a pneumatic extruder and a heating bed. For the preparation of 3D-printed matrices, only PCL was selected due to its lower melting temperature (*T_m_* = 70 °C) with respect to PDLLA (*T_m_* = 170 °C). To compare the results of degradation and release of *closo*-borate for films and 3D-matrices, a PCL-based material with 5 wt% Na[B_12_H_11_NH_3_] as filler was made. Before 3D printing, a composite was made by blending *closo*-borate with melted polymer.

A TGA-DSC analysis (Figure S7, Supplementary Materials) for Na[B_12_H_11_NH_3_]·2H_2_O revealed its suitability for 3D printing. The loss of two solvated water molecules in both air atmosphere and argon was observed when the sample was heated to 120–130 °C. Further heating to 300 °C resulted in the destruction of 2% CB in argon and 5% in air. Thus, this *closo*-borate showed to have a thermal stability suitable for 3D printing for most biodegradable polyesters based on aliphatic hydroxycarbonic acids.

**Figure 7:**
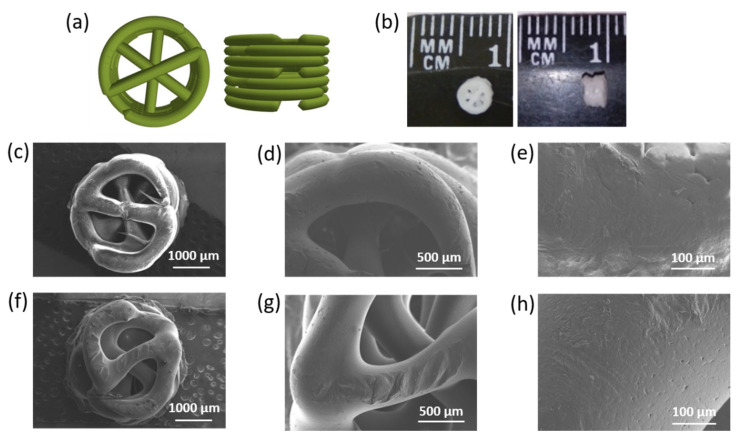
3D-printed matrices and their composites with *closo*-borate: (**a**) 3D model of the matrix (top and side views, respectively); (**b**) images of 3D-printed PCL/Na[B_12_H_11_NH_3_] matrix containing 5 wt% of the filler (top and side views, respectively); SEM images of the PCL matrix at different magnification: ×40 (**c**), ×100 (**d**), ×500 (**e**), and PCL/Na[B_12_H_11_NH_3_] composite containing 5 wt% of the filler at different magnification: ×40 (**f**), ×100 (**g**), ×500 (**h**).

The 3D model of the specimen represented a porous cylinder with an overall height of 2.5 mm and a diameter of 3.2 mm (Figure 7a,b). The specimen was made up of 7 layers of 0.36 mm in height each. 3D printing conditions were optimized for uniform formation of each layer and obtaining macroporous structures (see Section 2.7 for details). Taking into account the diameter of the printhead nozzle (0.4 mm), the theoretical volume of the material to print one specimen was 0.0075 cm^3^, the external geometric volume was 0.0203 cm^3^, the pore volume was 0.0128 cm^3^, and the porosity was 63%. The weight of the printed specimens was 11.9 ± 0.9 mg.

PCL composite matrices printed according to the specified 3D model were characterized by a homogeneous and similar morphology. The presence of pores in polymer filaments was the same as in film materials. Moreover, the spaces between the connected filaments provided interpenetrating macropores (Figure 7c–h). No filler aggregates were detected on or near the surface of the composites. The diameter of the 3D-printed matrices appeared to be slightly larger (about 4 mm) than it was programmed, while the filament thickness was close to the theoretical one.

Morphology, porosity, pore size, and permeability are important for adhesion, cell proliferation, vascularization, mineralization, and continuous tissue ingrowth. It has been reported that the formation of mineralized bone tissue can occur when pore sizes are larger than 200 μm, but pores and their connecting channels should be at least 300 μm in diameter to ensure sufficient vascularization (vascular sprouting) [49]. At the same time, proper cell multiplication can be achieved with pore sizes of no more than 1000 μm [50]. There are no definitive requirements for porosity, but it is known to be above 50% for satisfactory bone growth and vascularization [49,50]. The pore size and porosity of the obtained 3D-printed matrices were within the specified limits, and printing each subsequent layer with an axial shift of 60° allowed the presence of interpenetrating macropores. Thus, the 3D-printed composites meet the requirements necessary for their use as materials for bone tissue regeneration.

### 3.5. 3D-Printed Composites Filled with Hydrogel

The use of the *closo*-borate as a filler in a polymer matrix makes it possible to achieve rapid release and post-operative removal of tumor tissues by BNCT, if necessary. In this work, Na[B_12_H_11_NH_3_] was tested as a model *closo*-borate for the fabrication of 3D-printed materials. However, to ensure the required selective accumulation in cancer cells, the amino-containing cluster has to be modified with biofunctional linkers to ensure its targeted delivery. At the same time, the application of high temperatures during 3D printing can lead to the degradation of the biomolecule-modified *closo*-borate. To provide more gentle conditions avoiding the application of high temperatures, another approach for obtaining 3D composites containing *closo*-borate has been tested. In particular, CB was dispersed not in the matrix of the 3D-printed polymer scaffold, but in the hydrogel filling its macropores. Such an approach can be realized in an aqueous medium with heating not exceeding 50 °C.

In this study, a composite hydrogel was prepared with two natural biocompatible polymers, namely sodium alginate and gelatin [51,52]. Alginate is a non-allergenic polysaccharide widely used in the food industry and pharmaceutical products (scaffolds, drug delivery systems, etc.) [51], whereas gelatin is a product of partial hydrolysis of bone tissue collagen, which is characterized by lower antigenicity compared to native collagen [53]. Moreover, gelatin is a peptide containing the RGD (Arg-Gly-Asp) sequence, which promotes adhesion, proliferation, differentiation of cells, the formation of an extracellular matrix similar to bone, the prevention of apoptosis and, consequently, the acceleration of bone repair [54]. In addition, the presence of Ca^2+^ ions in the system facilitates the processes of osseointegration and bone regeneration [55].

**Figure 8:**
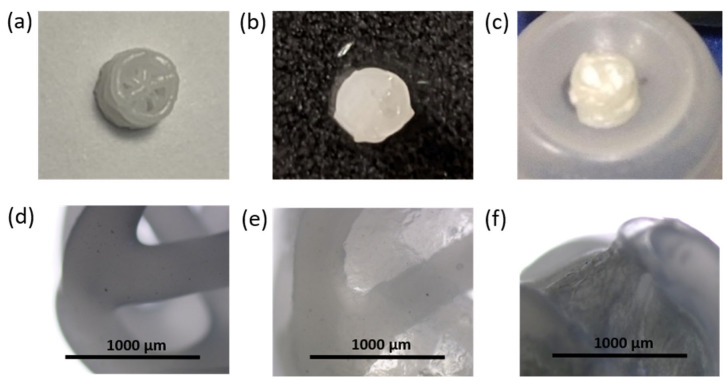
Photos and optical microscopy images (×4) of 3D-printed PCL matrices before (**a**,**d**) and after formation of the hydrogel loaded with Na[B_12_H_11_NH_3_] (**b**,**e**), as well as composite matrix after lyophilization (**c**,**f**).

In order to form a hydrogel layer at the scaffold surface and in its macropores, the printed 3D matrix was immersed into the aqueous viscous solution of sodium alginate and gelatin in a ratio of 25/75% (wt/wt) also containing Na[B_12_H_11_NH_3_] (5 wt%). After that, CaCl_2_ (1.13 wt%) was added to the system to crosslink sodium alginate and produce a hydrogel. The weight of the obtained undried composite 3D matrices filled with hydrogel was 22.4 ± 0.8 mg, of which hydrogel accounted for 10.5 ± 0.7 mg. Images of the obtained composites are shown in Figure 8.

The composite hydrogel was found to be homogenous and non-fluid; a picture of the free hydrogel is shown in Figure S8 (Supplementary Materials). The pores of the three-dimensional matrices were filled evenly (Figure 8), and the hydrogel did not flake or fall out of the matrix even after lyophilization. A study of the kinetics of re-swelling for *closo*-borate-containing hydrogel revealed that the specimen absorbed water quite quickly. After 30 min, the re-swelling degree (see Section 2.8) reached 99 ± 3%, and after the next 90 min was 104 ± 4%. After 5 days, the degree of re-swelling reached 126 ± 6%. Thus, these composite materials can be used both immediately after manufacturing and after storage in the dry state due to the preservation of their integrity and rapid re-swelling kinetics. This is important, for example, in the case of the need to postpone surgery and store the material.

### 3.6. Mechanical Properties of Composite Materials

#### 3.6.1. Composite Films

The mechanical properties of the films were studied by the uniaxial tension test at room temperature. The following characteristics were determined as a result of the tests: elastic modulus (Young’s modulus, *E*), yield strength (*σ_y_*), ultimate tensile strength (*σ_b_*), and elongation at break (*ε_b_*).

**Figure 9:**
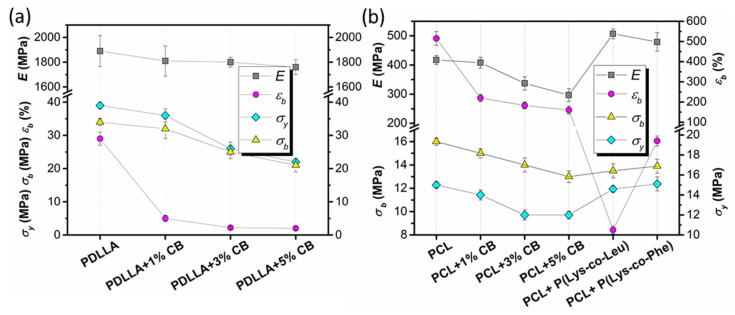
Tensile properties of the pristine PDLLA (**a**) and PCL (**b**) films and their composites with CB (Na[B_12_H_11_NH_3_]) and polypeptides (10 wt%).

Pristine PCL/P(Lys-*co*-Leu) and PCL/P(Lys-*co*-Phe), as well as PDLLA and PCL composites filled with 1, 3, or 5 wt% of Na[B_12_H_11_NH_3_] were examined. The averaged results are summarized in Figure 9, and the deformation curves of the materials are shown in Figure S9 (Supplementary Materials).

The initial part of stress-strain curves for the composite films was almost identical to that obtained for the pristine PCL film (Figure S9, Supplementary Materials). The values of the elastic modulus of the composites were slightly higher than Young’s modulus of the PCL film. At the same time, the transition from PCL to PCL/polypeptide composites led to a sharp decrease in the ultimate strain of the material until fracture. However, in the case of PCL/P(Lys-*co*-Phe), the ultimate strain to failure is about 2 times higher than this parameter for PCL/P(Lys-*co*-Leu). Apparently, the formation of composite material leads to sharp heterogenization of its structure and to the formation of structural defects, which serve as stress concentrators during the deformation.

The introduction of a filler Na[B_12_H_11_NH_3_] into both polymers led to a decrease in the stiffness and ultimate deformation of the materials. The obtained films based on PDLLA were stiffer than those based on PCL, which corresponds to the literature data [56], and the yield stresses for PDLLA were 2.0–2.6 times higher. They were also characterized by a more stable process of plastic deformation, as evidenced by the type of deformation curves. However, they collapsed at strains less than 30%.

Deformation curves of pure PCL films and PCL/Na[B_12_H_11_NH_3_] composites proved the heterogeneous structure of these materials. The tensile process of all PCL composites was realized through plastic deformation: after the initial “quasi-linear” segment of deformation (at deformations above 5–6%), local deformation acts were observed in separate parts of the specimens (an unstable character of the “stress-strain” relation with constant variations in the stress magnitude during tensile process). The increase in the content of the *closo*-borate was accompanied by a deterioration in the mechanical properties of the materials. However, the trend of changes was different for PDLLA- and PCL-based materials. The least significant decrease in modulus of elasticity as compared to material without filler was observed for PDLLA-based films (decrease to ~7%), whereas for PCL-based films, the same parameter decreased more dramatically (decrease to ~29%). At the same time, other parameters for PDLLA materials decreased more significantly as compared to the control material. Nevertheless, they exceeded the corresponding values for PCL-based materials except for the value of ultimate strain. Despite the decrease in tensile strength and elongation at break compared to the control materials, all the obtained compositions based on both PDLLA and PCL with CB have acceptable mechanical properties and are potentially suitable for use in regenerative medicine [50]. Materials based on PCL + 10 wt% P(Lys-*co*-Leu) and PCL + 10 wt% P(Lys-*co*-Phe) can also be used as the basis of a scaffold for bone regeneration.

#### 3.6.2. 3D-Composites

**Figure 10:**
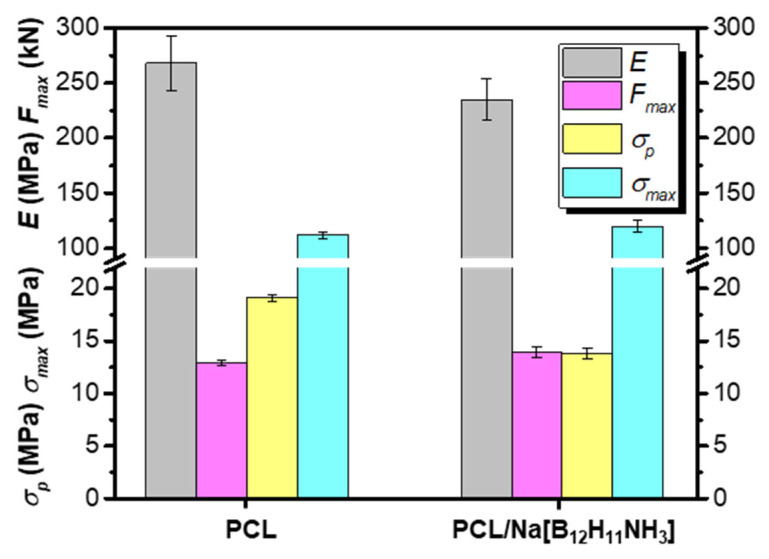
Compression properties of pure 3D cylindrical non-porous PCL matrix and PCL composite of the same shape containing 5 wt% Na[B_12_H_11_NH_3_].

The mechanical properties of the materials aimed to be used for bone tissue regeneration are extremely important since they have to support osteogenesis [57,58]. The normal functioning of bone tissue involves high compressive stresses. Therefore, the investigation of the mechanical properties of the composites is a very important step. To evaluate the effect of the *closo*-borate as a filler, PCL and PCL/Na[B_12_H_11_NH_3_] cylindrical specimens were studied in the compression test. The non-porous specimens were prepared by hot pressing. The tests were performed at room temperature and a compression speed of 10 mm/min. Young’s modulus, the force applied at the maximum compression (*F_max_*), the yield stress (*σ_p_*), and the compressive strength (*σ_max_*) were determined at a compression to the deformation to failure limit of 80%. The obtained results are shown in Figure 10. The deformation curves can be found in Figure S9 (Supplementary Materials).

From the results obtained, it is possible to suppose a plastic character of the deformation with a high strain life (no beginning of fracture of a specimen in compression up to 80%). The introduction of the filler into the matrix of PCL led to a reduction of stiffness of the material (small reduction of the modulus of elasticity and the yield stress) but practically was not reflected in the value of strength. Thus, the composite demonstrated properties acceptable for bone tissue regeneration, for example, for repairing trabecular bone defects at various anatomical locations [50,59].

### 3.7. Release of Closo-Borates and Matrix Degradation

#### 3.7.1. Composite Films

**Figure 11:**
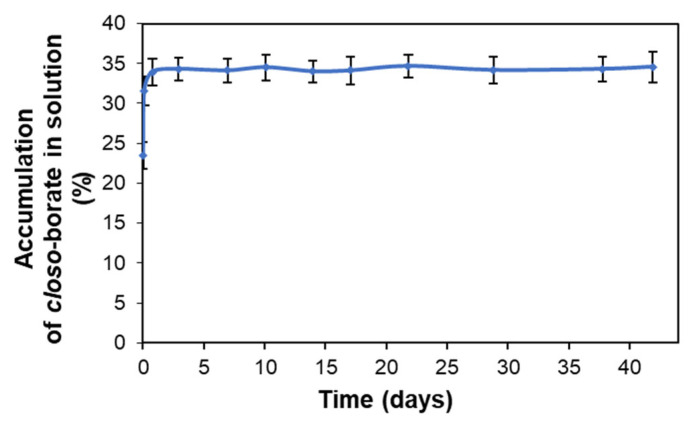
Desorption curve of Na_2_[B_10_H_9_OH] from the surface of PCL/P(Lys-*co*-Leu) in model solution (0.01M PBS, pH 7.4, 0.5 mL, 25 °C, ^11^B NMR spectroscopy).

The kinetics of *closo*-borate desorption from the surface of the PCL/P(Lys-*co*-Leu) films containing the biggest amount of the adsorbed Na_2_[B_10_H_9_OH] (adsorption with the use of crown ether) was studied. For this purpose, the specimens in the shape of disks (*D* = 15 mm) were placed in a solution simulating physiological conditions, namely, 0.01 M sodium phosphate buffer containing NaCl in the concentration of 0.15 mol/L (PBS, pH 7.4). The degree of *closo*-borate desorption was evaluated by determining the concentration of CB in solution using ^11^B NMR spectroscopy as described in Section 2.6. The dependence of *closo*-borate release on time is shown in Figure 11. The accumulation of CB in the model medium reached its maximum during the first day. After that, the amount of desorbed *closo*-borate did not change. Thus, the degree of desorbed Na_2_[B_10_H_9_OH] was 35 ± 2%, which is approximately equal to 13.9 ± 0.9 μg/cm^2^ (0.077 ± 0.004 μmol/cm^2^). The calculation was carried out according to the data on the adsorption capacity of the material obtained by ^11^B NMR spectroscopy (see Section 3.2.2).

**Figure 12:**
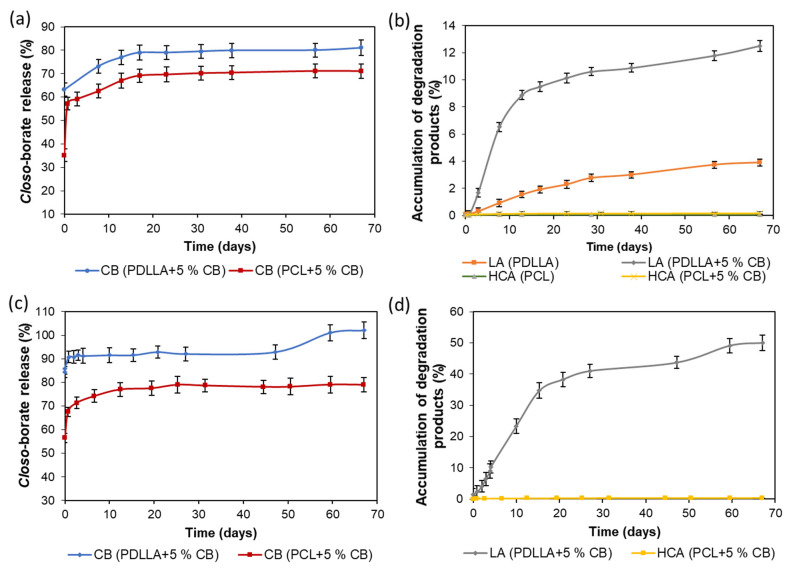
Profiles of CB (Na[B_12_H_11_NH_3_]) cumulative release from composite films (**a**,**c**) and accumulation of lactic and 6-hydroxycaproic acids (**b**,**d**) produced during degradation of PDLLA and PCL films obtained in 0.01 M PBS (**a**,**b**) and 0.1 M PBS (**c**,**d**). Conditions: 0.01 M PBS or 0.1 M PBS, pH 7.4, in presence of lipase, 37 °C, monitoring by ^1^H and ^11^B NMR spectroscopy.

In the case of composite films filled with *closo*-borate, the release was studied simultaneously with degradation. The investigation was carried out using PDLLA and PCL films filled with 5 wt% of Na[B_12_H_11_NH_3_]. Incubation of the specimens (10.7 ± 0.9 mg) was performed in 0.01 M PBS (pH 7.4) containing lipase, an enzyme active toward ester bonds, at 37 °C for 67 days. The release and biodegradation processes were monitored by ^11^B and ^1^H NMR spectroscopy, respectively, by adding boric acid (^11^B NMR) or DSS (^1^H NMR) as internal standards. The accumulation profiles for lactic acid (LA) and 6-hydroxycaproic acid (HCA), produced during hydrolysis of PDLLA and PCL, as well as Na[B_12_H_11_NH_3_] in solution, are shown in Figure 12 and Figure S10 (Supplementary Materials). Examples of ^1^H and ^11^B NMR spectra of the films’ degradation and release processes are shown in Figure S11 (Supplementary Materials).

At the initial step of degradation, a burst release of *closo*-borate was observed for both PDLLA- and PCL-based composites (Figure 12a). After 17 days, the release of CB reached its maximum for both composite films and remained practically unchanged during the following days. The total Na[B_12_H_11_NH_3_] release achieved in approximately 2 months was 82 and 71% for PDLLA and PCL composite films, respectively. The more effective release of *closo*-borate from PDLLA is in agreement with its faster degradation than in the case of PCL-based composites (Figure 12b). The observed burst release of CB in the first days, when the degradation has not yet begun, is a positive feature for the possibility of BNCT for residual tumor tissues immediately after surgery.

The degradation rate for PDLLA films with and without a filler was substantially different. In particular, the introduction of the filler led to a significant acceleration of material degradation. This fact can be related to an increase in the surface roughness of composites and, consequently, their specific surface area. As a result, the removal of the *closo*-borate from the surface and near-surface layers is more intense. The maximum of PDLLA degradation reached during about 2 months was approximately 12%. The degradation rate of PCL did not exceed 0.2%. Such feature of these polymers is known and explained by the higher crystallinity of PCL in contrast to amorphous PDLLA. Moreover, it is known that the degradation rate is also affected by the polymer molecular weight [47]. However, in our case, polymers used in the study had close molecular weight characteristics, and this factor cannot be considered for the explanation of degradation efficiency. In general, the slow degradation process can be caused by the inactivation of the enzyme due to acidification of the reaction medium provided by the accumulation over time of free LA and HCA.

To evaluate the effect of buffer capacity, the release and degradation of composites were additionally studied in the more concentrated buffer, namely 0.1 M PBS (pH 7.4), also containing lipase (Figure 12c,d). Indeed, increasing the buffer capacity accelerated the accumulation of lactic acid, and the degradation rate of PDLLA/Na[B_12_H_11_NH_3_] films reached about 35% within 14 days. After 2 months, the degradation rate was about 50%, which significantly exceeds the value obtained earlier using 0.01 M PBS. At the same time, the degradation of PCL-based composite within 2 months did not exceed 0.4% (Figure S10, Supplementary Materials). Despite the slight increase in the degradation rate detected, the overall process was too low and less affected by acidification than PDLLA. Thus, the most important issue in the long-term degradation of PCL is its high crystallinity.

In turn, the study of *closo*-borate accumulation in 0.1 M PBS also revealed a burst release at the initial stage of degradation. Already on the first day, about 91% of Na[B_12_H_11_NH_3_] was released from PDLLA films, and after 2 months, this value reached 100%. In the case of PCL, the release was 67 and 81% within the first day and after 2 months, respectively. Thus, the buffer capacity showed to affect both the release efficiency and degradation rate.

After completing the degradation process, the polymer films were extracted from the incubation medium, dried, and weighed. The obtained weight values of the specimens confirmed the degree of degradation established by NMR spectroscopy.

**Figure 13:**
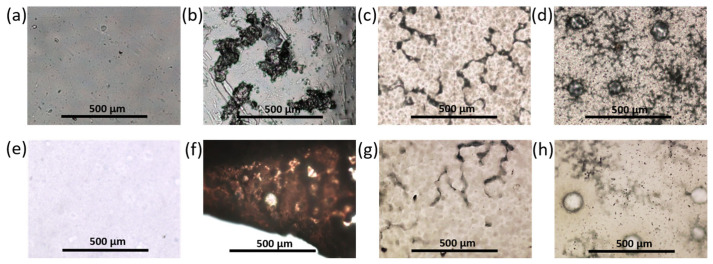
Optical microscopy of film surfaces (×10): PDLLA film without a filler before (**a**) and after (**e**) 2 months of degradation, PDLLA/Na[B_12_H_11_NH_3_] before (**b**) and after (**f**) 2 months of degradation, PCL film without a filler before (**c**) and after (**g**) 2 months of degradation, and PCL/Na[B_12_H_11_NH_3_] before (**d**) and after (**h**) 2 months of degradation. The filler content in all composites was 5 wt%.

The surface morphology of the unfilled and composite polymer films before and after degradation was examined by optical microscopy. Figure 13 shows images of control PDLLA and PCL films and composites with 5 wt% *closo*-borate before and after degradation with 0.1 M PBS to better visualize changes in the polymer matrix during degradation. A large number of CB aggregates were observed on the surface of PDLLA-based composite films before degradation, while the images during degradation showed a large number of lacunae of different sizes followed by the formation of large pores (Figure 13b,f). This is in agreement with the results of material degradation and release of CB. During degradation, the film became brittle, changed shape and lost transparency, and turned white (Figure S12a,b, Supplementary Materials). This is most likely due to the faster degradation of the amorphous part of the PDLLA with respect to a more stereoregular amorphous-crystalline phase, which remained in the film.

No significant changes in the structure and surface morphology of the PCL-based materials were observed visually or by microscopy. The presence of bubbles throughout the volume of the composite PCL materials was still observed (Figure 13d,h and Figure S12c,d in Supplementary Materials). Given the burst release of *closo*-borate and the slow degradation rate, it is possible to assume the localization of CB in the pores and “bubbles” of the PCL film.

#### 3.7.2. 3D-Printed Matrices

The study of matrix degradation was performed with the use of a more concentrated buffer solution (0.1 M PBS) in the presence of lipase at 37 °C. As in the previous case, monitoring of *closo*-borate release and accumulation of degradation products was performed using ^1^H and ^11^B NMR spectroscopy.

**Figure 14:**
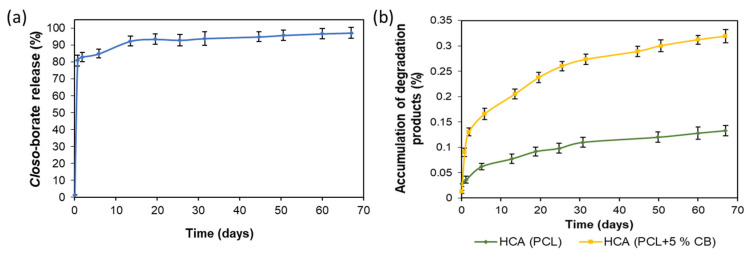
Profiles of cumulative release of CB (Na[B_12_H_11_NH_3_]) from 3D-printed PCL matrices (**a**) and degradation of 3D-printed PCL and PCL/Na[B_12_H_11_NH_3_] (**b**). Conditions: 0.1 M PBS, pH 7.4, in the presence of lipase, 37 °C, monitoring by ^1^H and ^11^B NMR spectroscopy. Composite contained 5 wt% of the filler.

Similar to the films, the release of the *closo*-borate had also burst character in the first days, reaching a plateau in the following days (Figure 14a). This can probably be explained by several factors: the porous structure of PCL materials (Figure 7) and the localization of *closo*-borate near the pore surface, as well as the large surface area from which the release occurs. In particular, 81% of CB was released on the first day, and after 2 months, it reached 97%.

The degradation of the pure PCL matrices was slower than for its composites with Na[B_12_H_11_NH_3_] (5 wt%) as in the case of the films (Figure 14b). This trend is in agreement with the results obtained by other research groups for composites based on aliphatic polyesters containing halloysite nanotubes, hydroxyapatite, and cellulose materials as the fillers [48,60,61,62]. Overall, the degradation over 67 days reached 0.14% for the pure PCL matrix and 0.32% for its composite.

The results on release efficiency and degree of degradation obtained with the use of NMR spectroscopy were consistent with the results obtained by gravimetry. According to the gravimetric data, mass loss was about 0.11 ± 0.01% for pure PCL and 5.1 ± 0.4% for PCL containing 5 wt% Na[B_12_H_11_NH_3_] as a filler.

Visual evaluation and examination of the morphology of the 3D-printed matrices after degradation using optical microscopy, as in the case of film materials, showed no significant changes (data not shown). The matrices retained their integrity, color, and shape.

**Figure 15:**
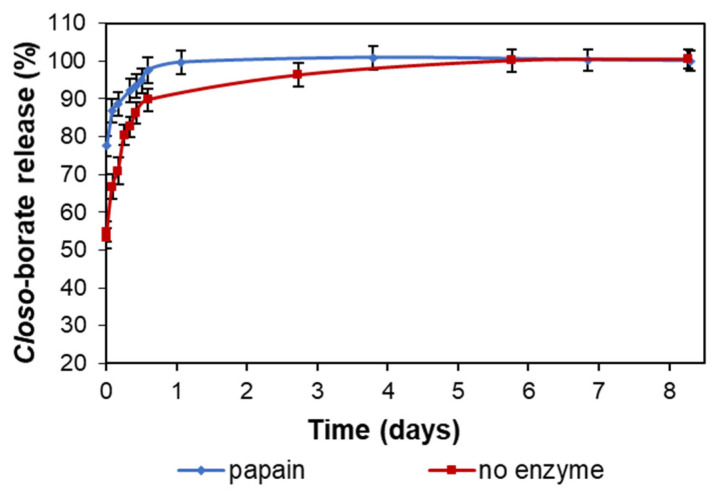
Profiles of cumulative release of Na[B_12_H_11_NH_3_] from alginate/gelatin hydrogel filling the pores of of 3D-printed PCL framework. Conditions: 0.1 M PBS, pH 7.4, with or without papain, 37 °C, monitoring by ^1^H and ^11^B NMR spectroscopy. Composite contained 5 wt% of the filler.

In addition, the release rate of *closo*-borate dispersed in the 3D polyester matrix was compared with the efficiency of CB release from the hydrogel covering the 3D polymer framework. To this aim, PCL porous matrix, filled with alginate/gelatin hydrogel containing Na[B_12_H_11_NH_3_], was freshly prepared (see Section 2.8), and the release of *closo*-borate was studied under the same conditions as for the 3D-printed composites without hydrogel. To promote the degradation of gelatin, a system containing papain, an enzyme with broad proteolytic activity, was added to the system. The results of the release of Na[B_12_H_11_NH_3_] from the hydrogel filling the pores of the 3D-printed framework with and without papain addition are presented in Figure 15.

It was found that a 100% release of CB was detected after a day in the presence of papain. Without papain, the same release efficiency was reached only after 6 days. At the same time, in the case of 3D-printed composites with the filler dispersed in the PCL matrix, 97% release was observed only after about 2 months.

**Figure 16:**
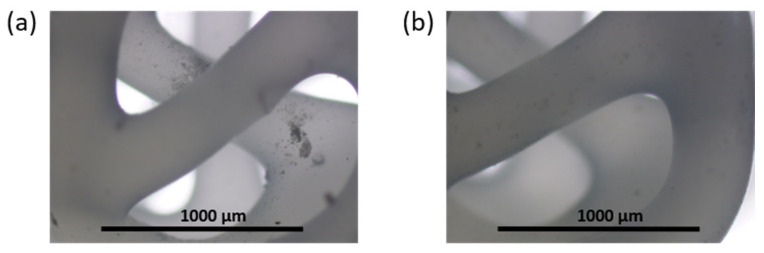
Optical microscopy (×4) of dried 3D-printed matrices contained alginate/gelatin hydrogel after the Na[B_12_H_11_NH_3_] release study without papain (**a**) and with papain (**b**).

The examination of matrix morphology at the end of the degradation process showed almost total removal of hydrogel from the 3D-printed PCL framework in both cases. Only a small amount of gel fragments was observed on the framework surface after washing and drying the specimen (Figure 16).

#### 3.7.3. Comparative Analysis of *Closo*-Borate Release from Composites

The data obtained in the study of CB adsorption/desorption and degradation of composites with the use of ^11^B NMR spectroscopy are summarized in Table 2. All types of obtained composite materials (both 2D and 3D) showed a burst release of CB on the first day of the study in a solution simulating physiological conditions. In the case of using the material with the largest amount of adsorbed CB, about 13–14 μg/cm^2^ was immediately desorbed. This is one-third of the content of the CB in the material. In the next 40 days, no changes were observed, which indicates a possible additional release of CB after a longer time. A prolonged effect can be achieved using films and three-dimensional matrices containing CB dispersed in aliphatic polyesters (PDLLA or PCL). The amount of released CB can be regulated by choosing the polymer base since, in the case of PCL, the initial release is slower than that for PDLLA. The use of alginate/gelatin hydrogel as a CB carrier resulted in its 100% release in the first days. This material is of interest in terms of the possibility of introducing various *closo*-borate loadings while maintaining the mechanical properties of the polymer framework. In this case, prolonged release of CB can be obtained by additional localization of *closo*-borate in the bulk of aliphatic polyester.

As was underlined in the Introduction, effective BNCT requires from 15–47 μg of ^10^B per 1 g of tumor tissue, which corresponds to 28–86 μg of CB per 1 g of tumor tissue when converted to the *closo*-borates studied in this work. Thus, all obtained composites can be used for effective BNCT, except for those containing surface-adsorbed *closo*-borate, since the content of CB in the material is quite low and difficult to control. The most promising is the use of composites with CB dispersed in the material both in polyesters and in a hydrogel or a combination of these approaches.

### 3.8. In Vitro Biological Evaluation

**Figure 17:**
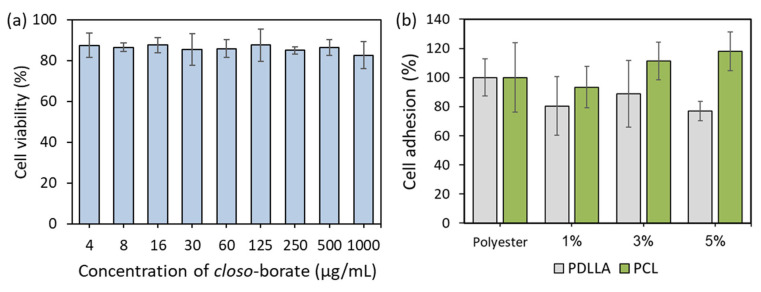
Viability of MG-63 cells (MTT, 72 h) in presence of Na[B_12_H_11_NH_3_] in solution (**a**) and their adhesion on the surface PDLLA/Na[B_12_H_11_NH_3_] and PCL/Na[B_12_H_11_NH_3_] films (**b**). Data presented as mean ± SD (*n* = 3).

The successful application of polymers and their composites as biomedical materials is determined by the absence of toxicity and cell adhesion on their surface. In vitro biocompatibility of the obtained materials was evaluated using human osteosarcoma cells (MG-63 cell line). The MTT test was performed for Na[B_12_H_11_NH_3_] solutions taken at different concentrations and for composites based on PDLLA and PCL with the *closo*-borate as a filler. The absence of cytotoxicity for Na[B_12_H_11_NH_3_] was shown over the entire range of concentrations tested (viability > 80%) (Figure 17a).

According to the data obtained, the PCL-based composites demonstrated very high cell adhesion (~100%), which did not depend on *closo*-borate content in the range of 1–5 wt% and was close to pure PCL material within an error (Figure 17b). In turn, a slight decrease in cell adhesion (up to 80–90%) was detected for PDLLA-based composites compared to pure PDLLA material (Figure 17b). Given that the viability of cells in the presence of CB in solution is about 80%, we can assume that the 80–90% viability of the adhered cells in the case of PDLLA-based composites may indirectly indicate that more *closo*-borate is localized on the material surface, in contrast to the PCL material. For PCL, taking into account the data on the study of the morphology of composites and the release profile of CB, most likely, the filler is located in the pores or thickness of the polymer matrix.

## 4. Conclusions

In the present work, various methods for obtaining polymer composites in the format of 2D- (film) and 3D- (matrix) materials containing *closo*-borates for BNCT were developed. PDLLA and PCL films containing different amounts of CB on the surface and in the bulk of the material were obtained by casting a polymer solution. The introduction of CB into the films or onto their surface was carried out by (1) dispersing 1, 3, and 5 wt% of CB in a polyester solution during film casting or (2) adsorption from solutions with different CB concentrations on the surface of preformed modified polymer films, respectively. The maximum adsorption capacity of CB (2.8–3.2 µg/cm^2^, according to ^11^B NMR spectroscopy, adsorption time 10 min) was achieved for *closo*-borates containing a hydroxyl substituent on the material surface with the largest amount of carboxyl and hydroxyl groups (partially hydrolyzed PDLLA). However, the use of crown ether capable of selectively capturing CB anion cluster counterion led to a significant increase in the adsorption degree of CB with the -OH group (39.6 ± 4.6 μg/cm^2^, according to ^11^B NMR spectroscopy, adsorption time 10 min) when aminated polyester was used. The obtained data are close to the results of the elemental analysis carried out using MP-AES.

For materials with dispersed CB, a slight decrease or retention at a similar level of mechanical properties was observed both in tension and in compression. In the case of composites and unfilled materials based on PDLLA, Young’s modulus, elongation at break, yield strength, and ultimate tensile strength varied within 1760–1890 MPa, 2–29%, 22–39 MPa, and 21–34 MPa, respectively. Similar parameters for PCL materials were 297–417 MPa, 160–515%, 12–15 MPa, and 13–16 MPa, respectively. In compression tests, Young’s modulus, yield stress, and compressive strength for the same non-porous PCL materials were 235–268 MPa, 14–19 MPa, and 112–120 MPa, respectively.

Three-dimensional macroporous matrices based on PCL with dispersed CB (5 wt%) and having pore characteristics that meet the requirements of bone tissue regeneration were fabricated using 3D printing by fused deposition modeling. As an alternative approach, unfilled analogous 3D-printed frameworks were filled with a CB-containing (5 wt%) alginate/gelatin hydrogel. For all types of obtained two- and three-dimensional materials, a burst release of *closo*-borates (67–100% for CB-filled materials and 33–35% for CB adsorbed in the presence of crown ether) was observed under simulated physiological conditions. The dispersion of CB in aliphatic polyester made it possible to provide a prolonged release of CB. Depending on the material, the subsequent release of CB (over 2 months) was 9–16%, while the use of alginate/gelatin hydrogel as a CB carrier provided 100% release in 1 day. The introduction of filler contributed to the acceleration of polyester material degradation in the model solution, and the maximum degradation rates after 2 months were about 50% for PDLLA and 0.4% for PCL, both containing 5 wt% CB. Thus, depending on the method of preparation and the polymer used, the rate of the matrix degradation and the CB release can be controlled. The mechanical properties of developed composite materials in tension and compression, the demonstrated biocompatibility with human osteosarcoma cells, and a sufficient level of CB release indicate promising applicability of these 2D and 3D materials in bone tissue regeneration and BNCT.

## Figures and Tables

**Table 1:** Results on adsorption of *closo*-borates on the surface of modified PDLLA and PCL materials.

	Polymer Film		*Closo*-Borate	Adsorption Capacity **		AE, %
Polymer	Modification	Functional Groups		µg/cm^2^	µmol/cm^2^	
PCL	10 wt% P(Lys-*co*-Leu)	-NH_2_	Na_2_[B_10_H_9_OH]	1.8	0.010	3.6
PCL	Surface hydrolysis	-COOH and -OH	Na_2_[B_10_H_9_OH]	1.6	0.009	3.1
PDLLA	Surface hydrolysis	-COOH and -OH	Na_2_[B_10_H_9_OH]	3.2	0.018	6.2
PDLLA	Surface hydrolysis	-COOH and -OH	Na[B_12_H_1__1_NH_3_]	1.3	0.007	2.6
PDLLA	Surface hydrolysis	-COOH and -OH	Na[B_10_H_9_NH=C(OH)CH_3_] *	2.8	0.014	4.8
PDLLA	Surface hydrolysis	-COOH and -OH	Na[B_10_H_9_NH=C(NH_2_)CH_3_] *	0.4	0.002	0.6

Conditions: A specimen: two disc-shaped films (*D* = 15 mm); adsorption time: 10 min; temperature: 25 °C; concentration of *closo*-borates: 4 µmol/mL; solution volume: 0.5 mL; solvent: D_2_O. * Used as a solvent D_2_O/CD_3_CN—92/8% (*v*/*v*). ** According to ^11^B NMR spectroscopy. Relative standard deviation ranged from 7–13%.

**Table 2:** Release of *closo*-borate from polymer composite containing both CB adsorbed on the surface and dispersed in the bulk of the material (according to ^11^B NMR spectroscopy).

Type of Composite	Material Containing CB	Amount of CB Released					
		1st Day		From 2 to 17 Days		From 47 to 67 Days	
		%	μg/cm^2 b^/μg/mg ^c^	%	μg/mg	%	μg/mg
Films with adsorbed CB ^a^	PCL/ P(Lys-*co*-Leu)	33–35	13–14	0	0	Not studied	
Films with dispersed CB	PDLLA	to 91	to 45	0	0	9	4
	PCL	to 67	to 33	11	6	3	1
3D-printed porous matrices with dispersed CB	PCL	to 81	to 41	12	6	4	2
3D-printed porous matrices filled with CB containing hydrogel	Alginate/ gelatin hydrogel	to 100	to 50 ^d^	0	0	0	0

^a^ Obtained in the presence of crown ether; ^b^ for films coated with adsorbed CB; ^c^ for all kinds of materials except films with adsorbed CB; ^d^ calculated per unit of freshly prepared hydrogel.

## Data Availability

Data are available within the article and its Supplementary Materials.

## Supplementary Materials

The supporting information can be downloaded at: https://www.mdpi.com/article/10.3390/polym14183864/s1, Figure S1: ^11^B NMR spectra of used in adsorption *closo*-borates: Na[B_12_H_11_NH_3_]·2H_2_O (a), Na_2_[B_10_H_9_OH]·2H_2_O (b), Na[B_10_H_9_NH=C(NH_2_)CH_3_]·2H_2_O (c), Na[B_10_H_9_NH=C(OH)CH_3_]·2H_2_O (d). The analysis was performed for a solution of compounds in CD_3_CN at 96.32 MHz for ^11^B, Figure S2: Scheme of the PDLLA surface hydrolysis and modification with ethylenediamine, Figure S3: Images of some PDLLA and PCL films before and after Na2[B10H9OH] adsorption (optical microscopy, ×4): PCL (image of a region with a higher degree of crystallinity) before adsorption (a), PCL with carboxyl and hydroxyl groups before (b) and after (h) adsorption, PCL-EDA after adsorption (c), PCL-PLys after adsorption (d), PCL/P(Lys-*co*-Leu) before adsorption (e), PCL/P(Lys-*co*-Phe) before adsorption (f) and PDLLA with carboxyl and hydroxyl groups after adsorption (g), Figure S4: SEM images of pristine and composite PCL/polypeptide films with different magnification (×100, ×500): PCL (a,d), PCL/P(Lys-*co*-Leu) (b,e) and PCL/P(Lys-*co*-Phe) (c,f), Figure S5: Examples of attenuated total reflection-Fourier transform infrared (ATR-FTIR) spectra of obtained film materials before and after adsorption of *closo*-borates on their surface in comparison with the ATR-FTIR spectrum of Na[B_12_H_11_NH_3_], Figure S6: SEM images of pristine and composite PDLLA and PCL films containing dispersed *closo*-borate with different magnification (×100, ×1000): PDLLA (a,e), PDLLA+5 wt% Na[B_12_H_11_NH_3_] (b,f), PCL (c,g) and PCL+5 wt% Na[B_12_H_11_NH_3_] (d,h), Figure S7: TGA-DSC curves for Na[B_12_H_11_NH_3_]: (a) argon atmosphere, (b) air atmosphere, Figure S8: Images of alginate-gelatin hydrogel loaded with 5 wt% Na[B_12_H_11_NH_3_]: photo (a,b) and optical microscopy (×4) (c), Figure S9: Tensile stress-strain curves for pristine and composite PDLLA and PCL films with Na[B_12_H_11_NH_3_] or polypeptide (a–c) and compression stress-strain curves for monolithic PCL sample and its composite with Na[B_12_H_11_NH_3_], Figure S10: Profiles of accumulation of 6-hydroxycaproic acid (HCA) produced during degradation of PCL-based composite films obtained in sodium phosphate buffer with 0.15 mol/L NaCl (PBS, pH 7.4) with different buffer capacity. Conditions: 0.01 M or 0.1 M PBS in presence of lipase, 37 °C, monitoring by ^1^H NMR spectroscopy, Figure S11: NMR spectra examples of degradation of PDLLA or PCL films filled with 5 wt% Na[B_12_H_11_NH_3_] in model physiological solution (pH 7.4, in presence of lipase, 37 °C): (a) release of *closo*-borate, (b) accumulation of lactic acid (from PDLLA) and (c) accumulation of 6-hydroxycaproic acid (from PCL). The analysis was performed with the use D^2^O as a solvent at 400.13 and 128.378 MHz for ^1^H and ^11^B/^11^B{^1^H}, respectively, Figure S12: Photos of film samples based on PDLLA + 5 wt% Na[B_12_H_11_NH_3_] before degradation (a), after 67 days of degradation (b) and based on PCL + 5 wt% Na[B_12_H_11_NH_3_] before degradation (c) and after 67 days of degradation (d). Conditions: 0.1 M PBS, 37 °C, in presence of lipase.
