# Supplementary material for: Design, Fabrication and Characterization of Biodegradable Composites Containing Closo-Borates as Potential Materials for Boron Neutron Capture Therapy
Source: Polymers (Basel). 2022 Sep 15;14(18):3864. doi: 10.3390/polym14183864 (PMC9506383; doi:10.3390/polym14183864)
Supplement: Supplementary file 1 [file polymers-14-03864-s001.zip › polymers-1866270-supplementary.pdf]

polymers

MDPI

Article

Design, Fabrication and Characterization of Biodegradable Composites Containing *Closo*-Borates as Potential Materials for Boron Neutron Capture Therapy

Mariia Stepanova[1,*], Anatoliy Dobrodumov[1], Ilia Averianov[1], Iosif Gofman[1], Juliya Nashchekina[2], Ivan Guryanov[3,*], Ilya Klyukin[4], Andrey Zhdanov[4], Evgenia Korzhikova-Vlakh[1,3] and Konstantin Zhizhin[4]

[1] Institute of Macromolecular Compounds, Russian Academy of Sciences, St. Petersburg 199004, Russia
[2] Institute of Cytology, Russian Academy of Sciences, St. Petersburg 194064, Russia
[3] Institute of Chemistry, Saint-Petersburg State University, St. Petersburg 198504, Russia
[4] Kurnakov Institute of General and Inorganic Chemistry, Russian Academy of Sciences, Moscow 119991, Russia
* Correspondence: maristepanova@hq.macro.ru (M.S.); ivan.guryanov1@gmail.com (I.G.)

Citations: Stepanova, M.; Dobrodumov, A.; Averianov, I.; Gofman, I.; Nashchekina, J.; Guryanov, I.; Klyukin, I.; Zhdanov, A.; Korzhikova-Vlakh, E.; Zhizhin, K. Design, Fabrication and Characterization of Biodegradable Composites Containing *Closo*-Borates as Potential Materials for Boron Neutron Capture Therapy. *Polymers* **2022**, *14*, 3864. https://doi.org/10.3390/polym14183864

Academic Editor: Beom Soo Kim

Received: 29 July 2022
Accepted: 13 September 2022
Published: 15 September 2022

Publisher's Note: MDPI stays neutral with regard to jurisdictional claims in published maps and institutional affiliations.

Copyright: © 2022 by the authors. Submitted for possible open access publication under the terms and conditions of the Creative Commons Attribution (CC BY) license (https://creativecommons.org/licenses/by/4.0/).

Supplementary Material

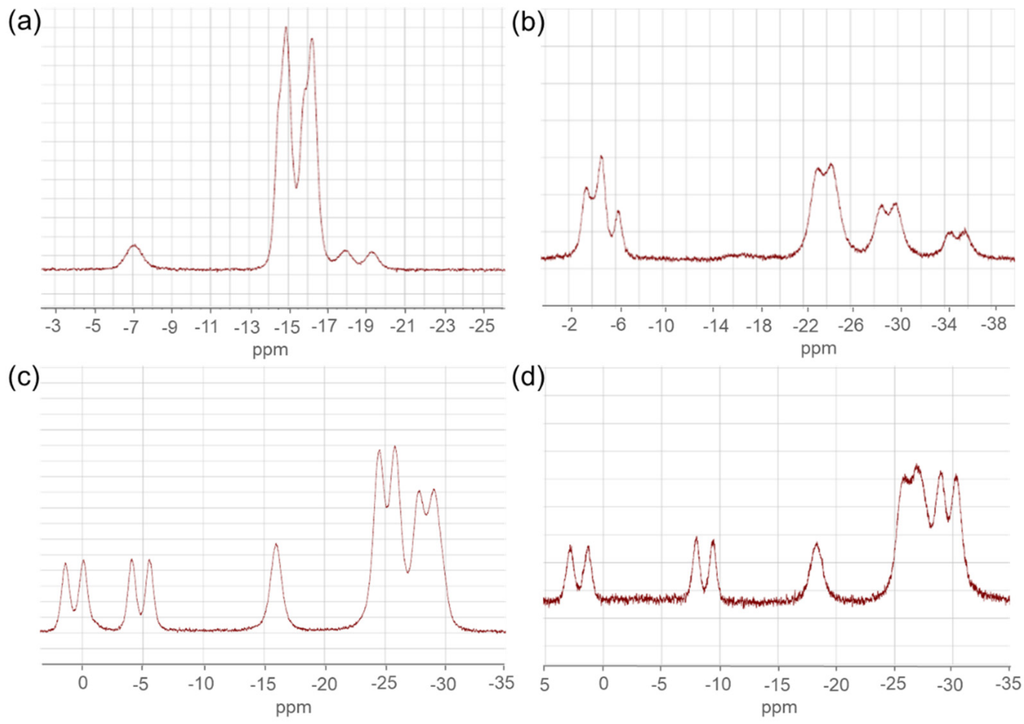

Figure S1. ^{11}B NMR spectra of used in adsorption *closo*-borates: $Na[B_{12}H_{11}NH_3]·2H_2O$ (a), $Na_2[B_{10}H_9OH]·2H_2O$ (b), $Na[B_{10}H_9NH{=}C(NH_2)CH_3]·2H_2O$ (c), $Na[B_{10}H_9NH{=}C(OH)CH_3]·2H_2O$ (d). The analysis was performed for a solution of compounds in CD_3CN at 96.32 MHz for ^{11}B.

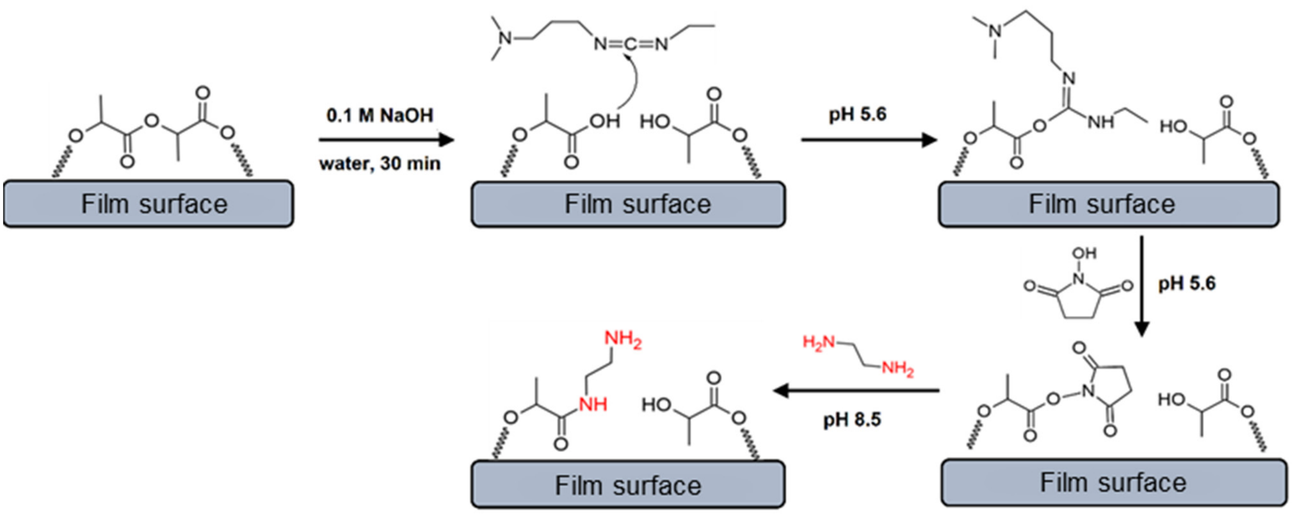

Figure S2. Scheme of the PDLLA surface hydrolysis and modification with ethylenediamine.

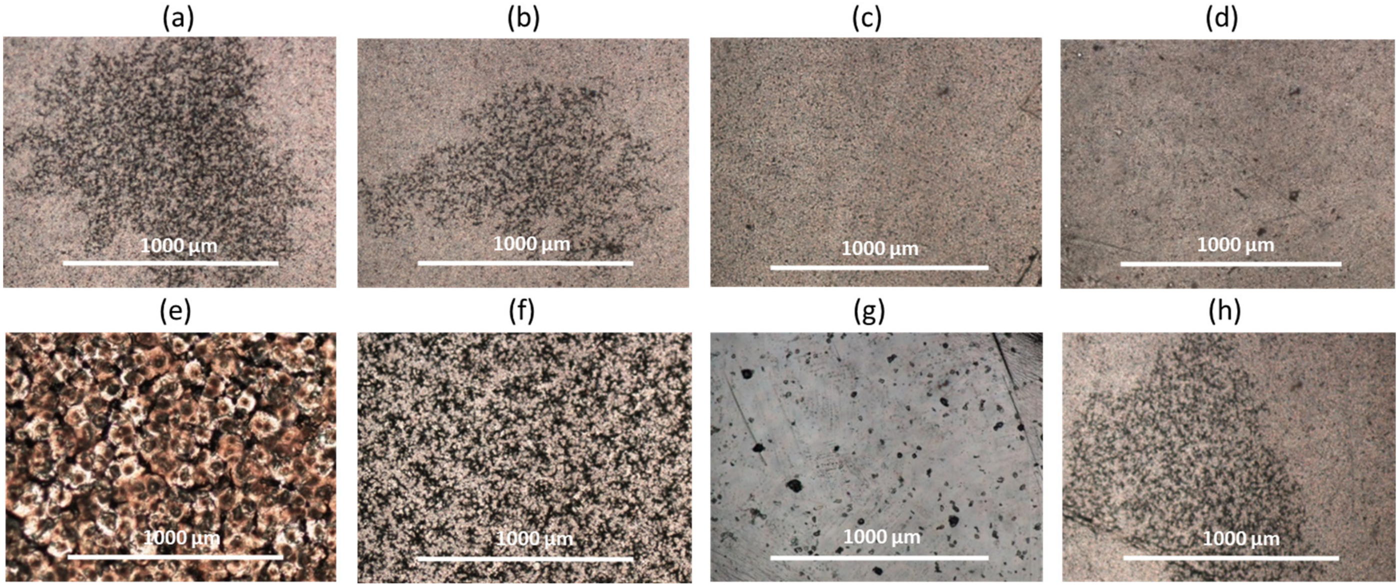

Figure S3. Images of some PDLLA and PCL films before and after $Na_2[B_{10}H_9OH]$ adsorption (optical microscopy, x4): PCL (image of a region with a higher degree of crystallinity) before adsorption (**a**), PCL with carboxyl and hydroxyl groups before (**b**) and after (**h**) adsorption, PCL-EDA after adsorption (**c**), PCL-PLys after adsorption (**d**), PCL/P(Lys-*co*-Leu) before adsorption (**e**), PCL/P(Lys-*co*-Phe) before adsorption (**f**) and PDLLA with carboxyl and hydroxyl groups after adsorption (**g**).

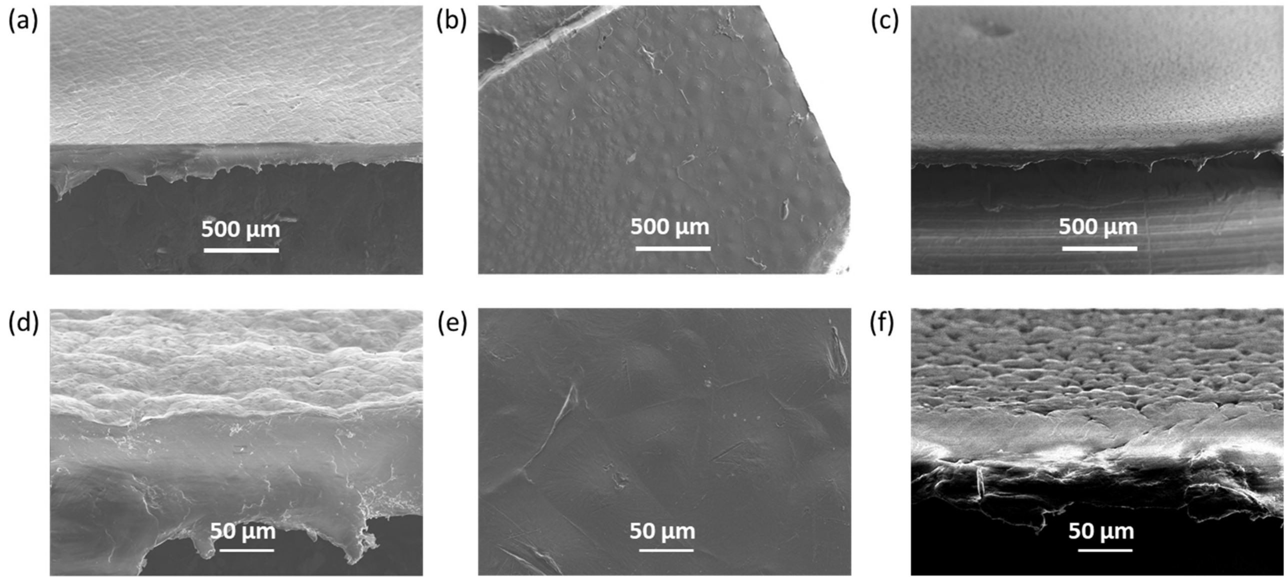

Figure S4. SEM images of pristine and composite PCL/polypeptide films with different magnification (x100, x500): PCL (**a**, **d**), PCL/P(Lys-*co*-Leu) (**b**, **e**) and PCL/P(Lys-*co*-Phe) (**c**, **f**).

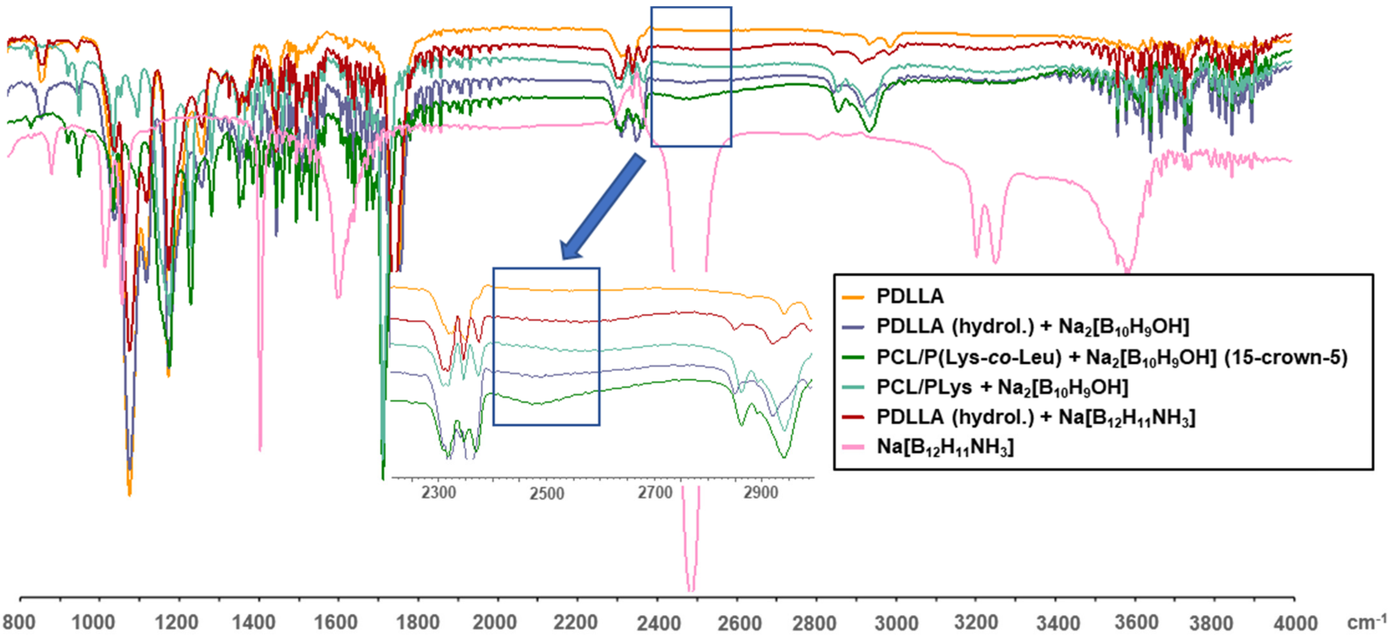

Figure S5. Examples of attenuated total reflection-Fourier transform infrared (ATR-FTIR) spectra of obtained film materials before and after adsorption of *closo*-borates on their surface in comparison with the ATR-FTIR spectrum of $Na[B_{12}H_{11}NH_3]$.

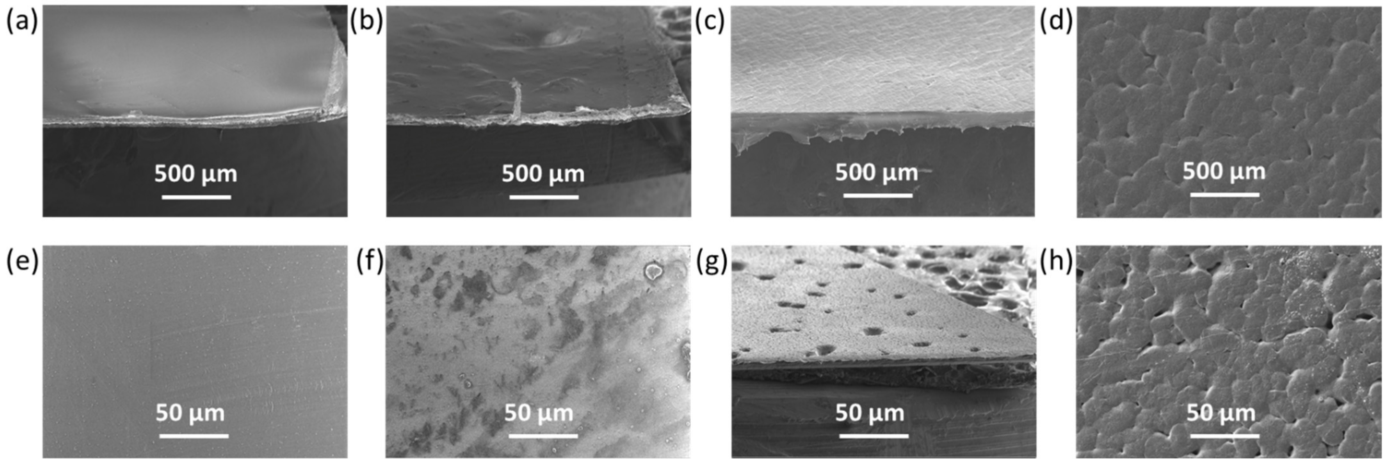

Figure S6. SEM images of pristine and composite PDLLA and PCL films containing dispersed *closo*-borate with different magnification (x100, x1000): PDLLA (**a**, **e**), PDLLA+5 wt% $Na[B_{12}H_{11}NH_3]$ (**b**, **f**), PCL (**c**, **g**) and PCL+5 wt% $Na[B_{12}H_{11}NH_3]$ (**d**, **h**).

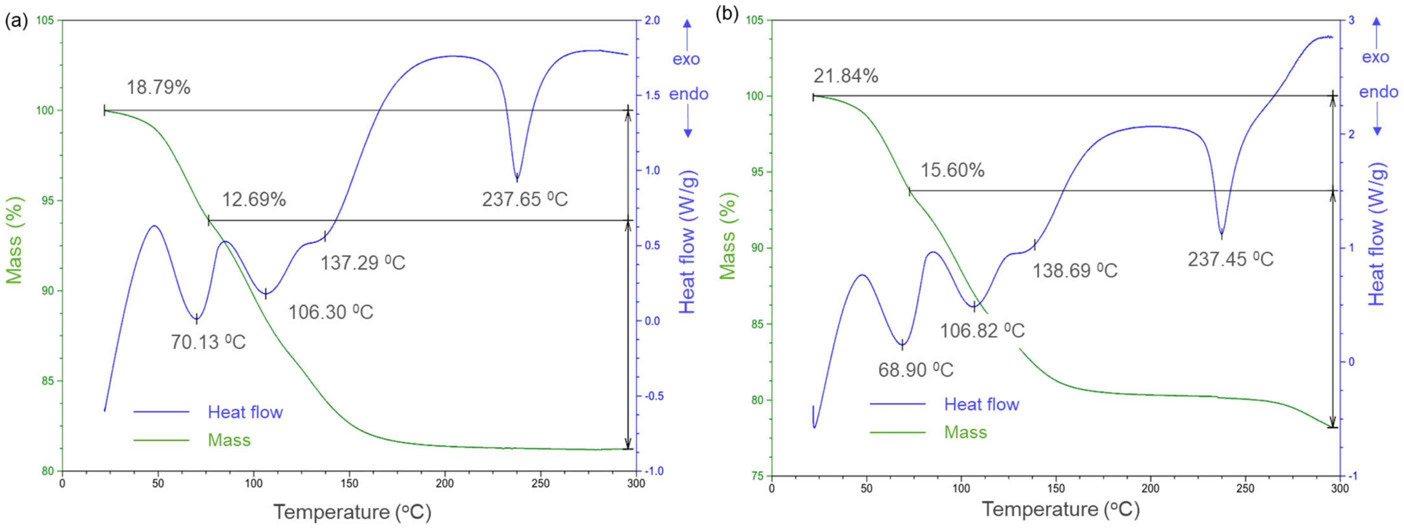

Figure S7. TGA-DSC curves for $Na[B_{12}H_{11}NH_3]$: (**a**) argon atmosphere, (**b**) air atmosphere.

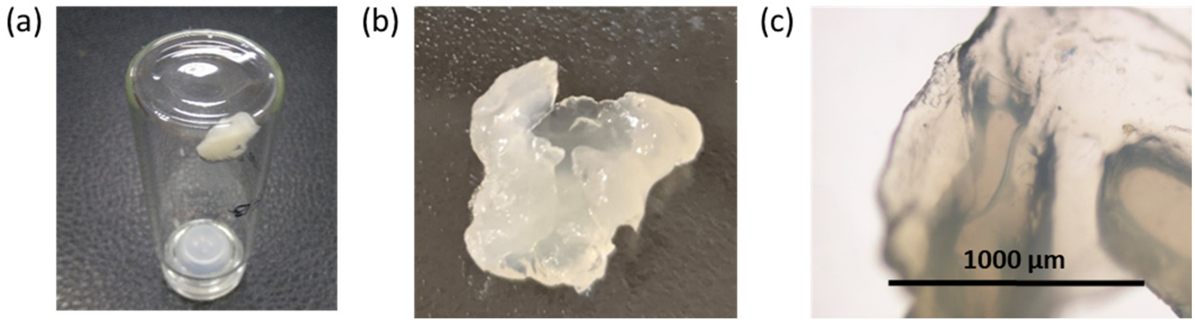

Figure S8. Images of alginate-gelatin hydrogel loaded with 5 wt% $Na[B_{12}H_{11}NH_3]$: photo (**a**, **b**) and optical microscopy (×4) (**c**).

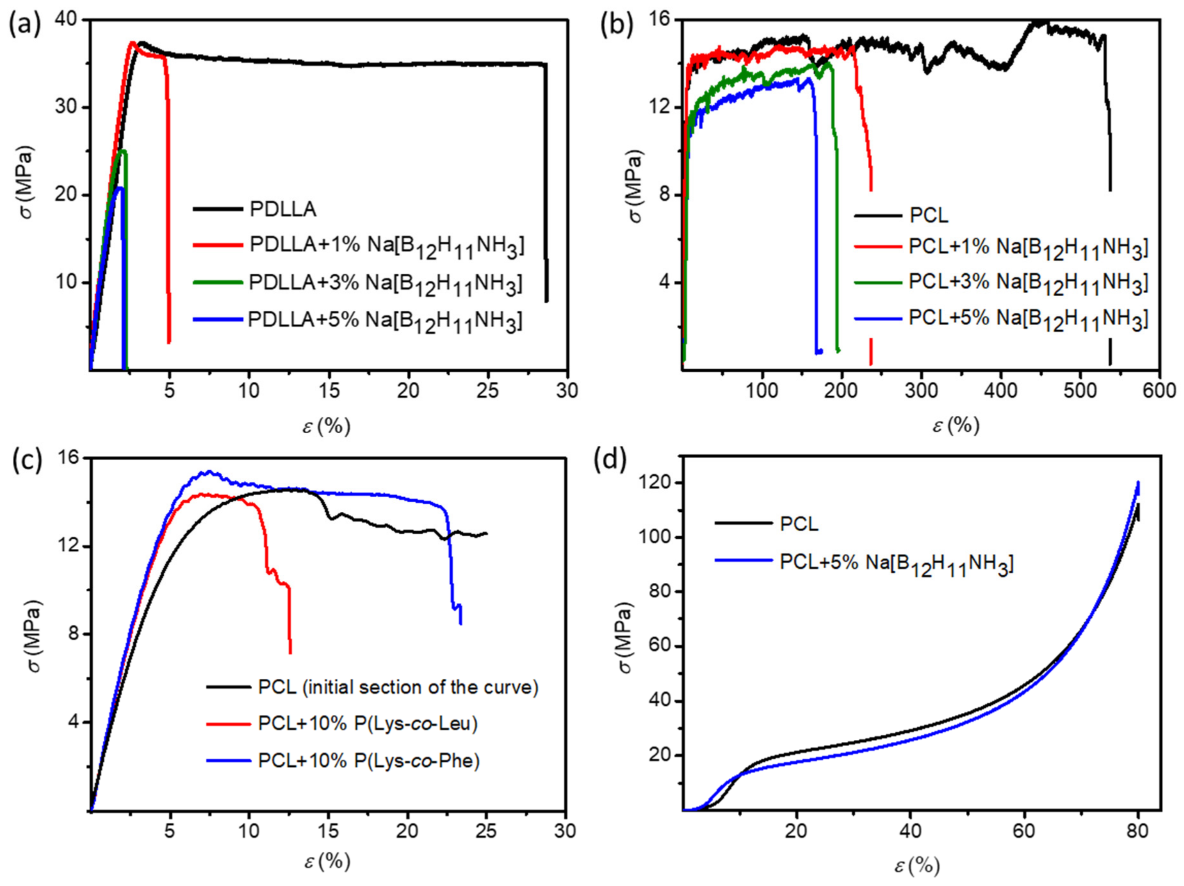

Figure S9. Tensile stress-strain curves for pristine and composite PDLLA and PCL films with Na[$B_{12}H_{11}NH_3$] or polypeptide (**a**-**c**) and compression stress-strain curves for monolithic PCL sample and its composite with Na[$B_{12}H_{11}NH_3$].

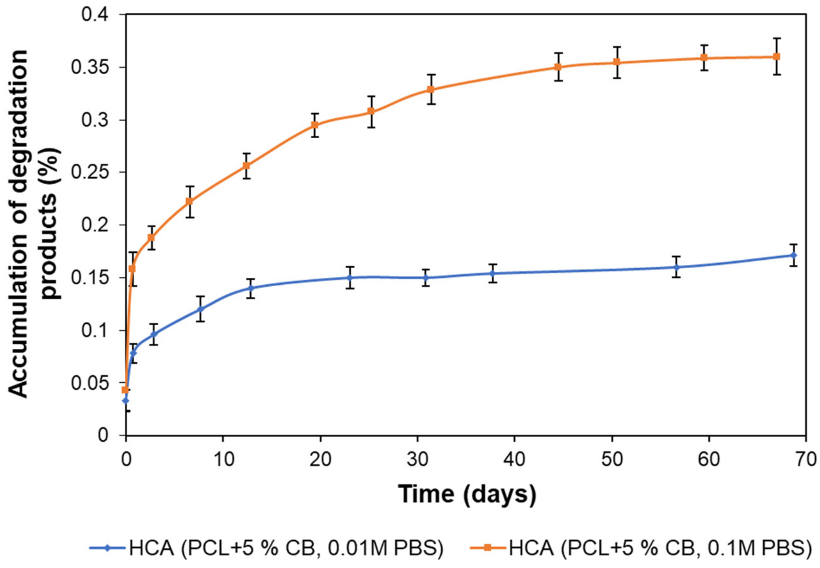

Figure S10. Profiles of accumulation of 6-hydroxycaproic acid (HCA) produced during degradation of PCL-based composite films obtained in sodium phosphate buffer with 0.15 mol/L NaCl (PBS, pH 7.4) with different buffer capacity. Conditions: 0.01 M or 0.1 M PBS in presence of lipase, 37 °C, monitoring by ^{1}H NMR spectroscopy.

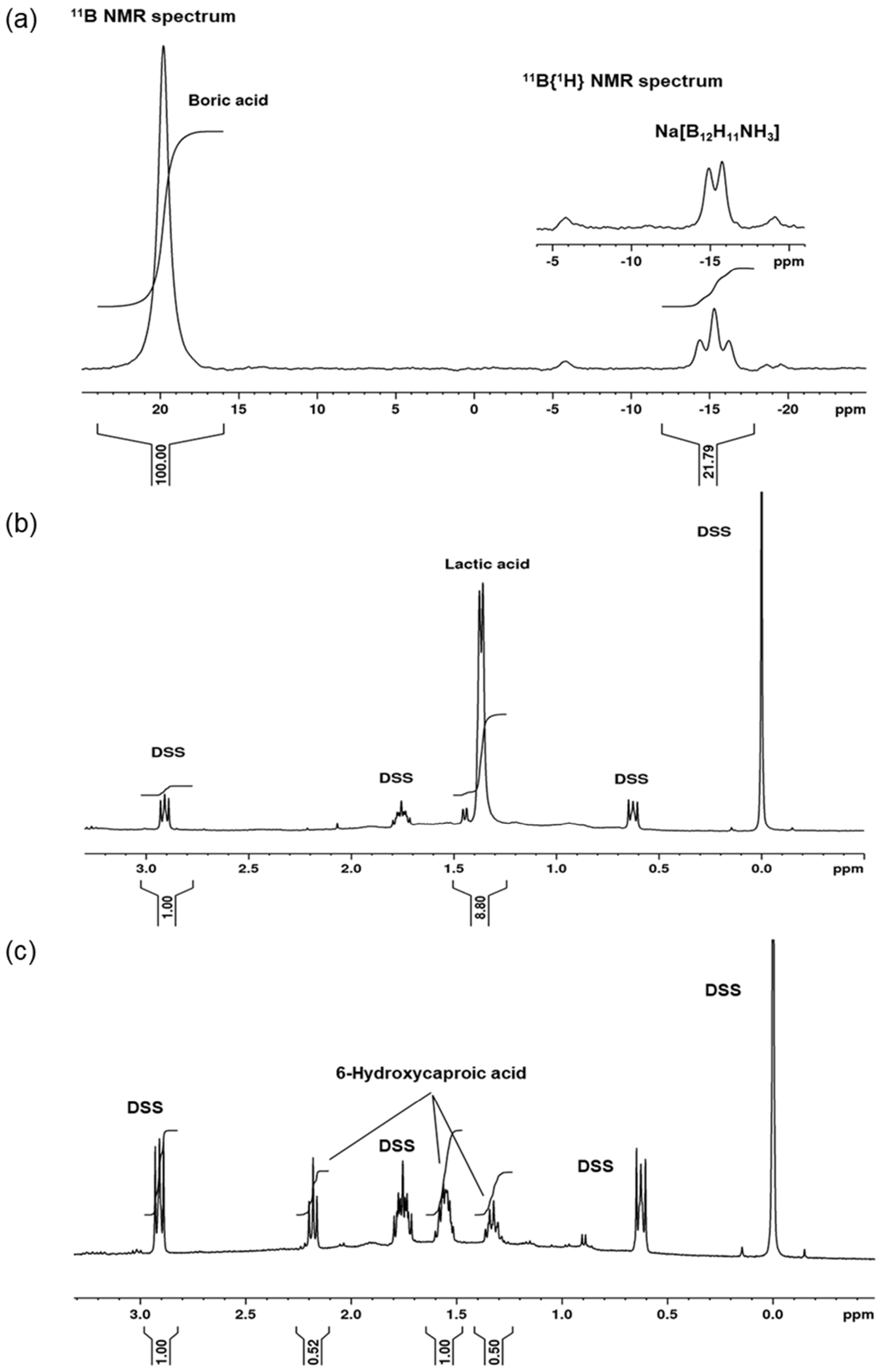

Figure S11. NMR spectra examples of degradation of PDLLA or PCL films filled with 5 wt% $Na[B_{12}H_{11}NH_3]$ in model physiological solution (pH 7.4, in presence of lipase, 37 °C): (**a**) release of *closo*-borate, (**b**) accumulation of lactic acid (from PDLLA) and (**c**) accumulation of 6-hydroxycaproic acid (from PCL). The analysis was performed with the use D_2O as a solvent at 400.13 and 128.378 MHz for ^1H and $^{11}B/^{11}B\{^1H\}$, respectively.

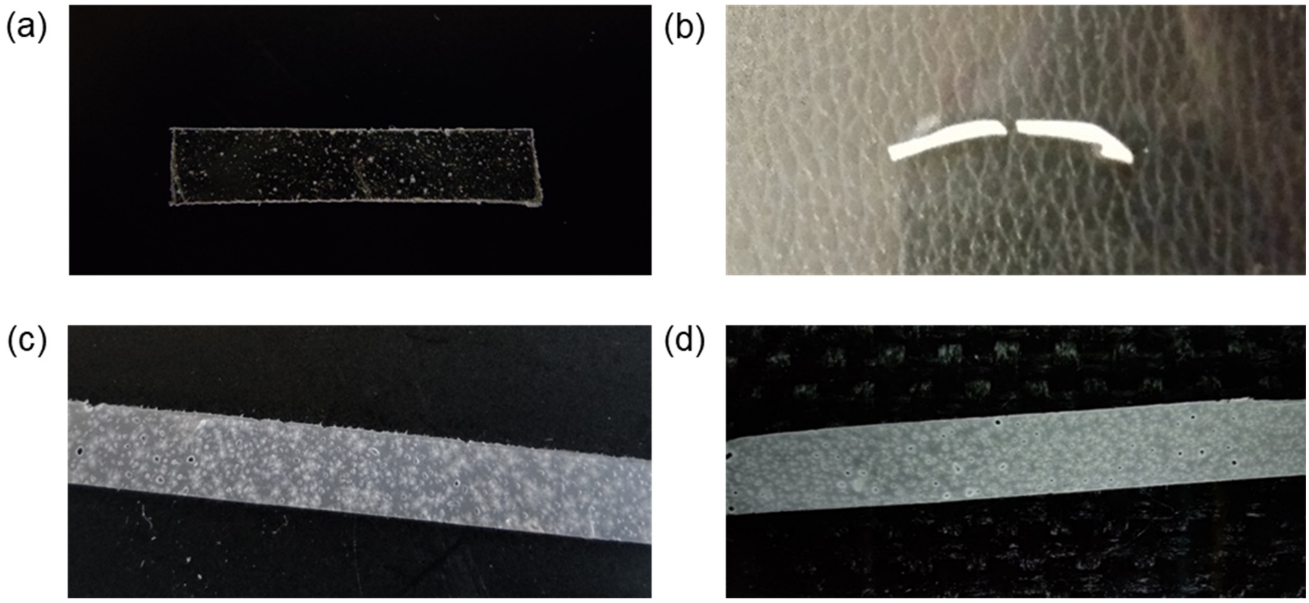

Figure S12. Photos of film samples based on PDLLA + 5 wt% Na[$B_{12}H_{11}NH_3$] before degradation (**a**), after 67 days of degradation (**b**) and based on PCL + 5 wt% Na[$B_{12}H_{11}NH_3$] before degradation (**c**) and after 67 days of degradation (**d**). Conditions: 0.1 M PBS, 37°C, in presence of lipase.
